# TRPC6 is a mechanosensitive channel essential for ultrasound neuromodulation in the mammalian brain

**DOI:** 10.1073/pnas.2404877121

**Published:** 2024-12-03

**Authors:** Yumi Matsushita, Kaede Yoshida, Miyuki Yoshiya, Takahiro Shimizu, Satoshi Tsukamoto, Nobuki Kudo, Yuichi Takeuchi, Makoto Higuchi, Masafumi Shimojo

**Affiliations:** ^a^Advanced Neuroimaging Center, National Institutes for Quantum Science and Technology, Chiba 263-8555, Japan; ^b^Department of Biopharmaceutical Sciences and Pharmacy, Faculty of Pharmaceutical Sciences, Hokkaido University, Sapporo 060-0812, Japan; ^c^Laboratory Animal and Genome Sciences Section, National Institutes for Quantum Science and Technology, Chiba 263-8555, Japan; ^d^Division of Bioengineering and Bioinformatics, Laboratory of Biomedical Engineering, Faculty of Information Science and Technology, Hokkaido University, Sapporo 060-0814, Japan; ^e^Laboratory of Pharmacotherapy, Department of Pharmacy, Faculty of Pharmacy, Kindai University, Osaka 577-8502, Japan

**Keywords:** ultrasound, mechanosensitive channels, TRPC6, neuromodulation, cortical neuron

## Abstract

Ultrasound neuromodulation has become an innovative technology that enables noninvasive intervention in mammalian brain circuits with high spatiotemporal precision. Despite the expanding utility of ultrasound neuromodulation in the neuroscience research field and clinical applications, the molecular and cellular mechanisms by which ultrasound impacts neural activity in the brain are still largely unknown. Here, we report that transient receptor potential canonical 6 (TRPC6), a mechanosensitive nonselective cation channel, is essential for ultrasound neuromodulation of mammalian neurons in vitro and in vivo. We first demonstrated that ultrasound irradiation elicited rapid and robust Ca^2+^ transients mediated via extracellular Ca^2+^ influx in cultured mouse cortical and hippocampal neurons. Ultrasound-induced neuronal responses were massively diminished by blocking either the generation of action potential or synaptic transmission. Importantly, both pharmacological inhibition and genetic deficiency of TRPC6 almost completely abolished neuronal responses to ultrasound. Furthermore, we found that intracerebroventricular administration of a TRPC6 blocker significantly attenuated the number of neuronal firings in the cerebral cortex evoked by transcranial ultrasound irradiation in mice. Our findings indicate that TRPC6 is an indispensable molecule of ultrasound neuromodulation in intact mammalian brains, providing fundamental understanding of biophysical molecular mechanisms of ultrasound neuromodulation as well as insight into its future feasibility in neuroscience and translational research in humans.

## Significance

In this study, we investigated the detailed biological mechanism of ultrasound neuromodulation in the mammalian brain. Taking advantage of ultrasound devices compatible with both neuronal cultures and brains, we systematically characterized the intrinsic properties of cellular responses to ultrasound in mouse cortical neurons and evaluated whether the findings in both in vitro and in vivo experiments were consistent with each other. Most strikingly, this approach eventually identified a specific mechanosensitive ion channel named TRPC6 as a key biosensor essential for neuronal responses to ultrasound neuromodulation in the intact mammalian brain. Our findings have provided fundamental information for the development of advanced ultrasound neuromodulation in humans.

Neuromodulation is a cardinal technique for altering neuronal activity in the brain circuit by delivering various external stimuli such as electrical currents, magnetic field, and acoustic irradiation. Such approaches enable noninvasive, nonsurgical intervention in the neuronal activity of targeted tissue in order to understand brain function in basic neuroscience research as well as to therapeutically ameliorate brain dysfunctions in neurological disorders (1, 2). For instance, transcranial direct current stimulation and transcranial magnetic stimulation have recently been established as neuromodulation techniques that can alter the neocortical activity of the human brain, and they have provided considerable benefit to therapeutical applications for epilepsy, depression, and disorders of consciousness (3). However, these conventional modalities affect a relatively broad range of the brain volume and are not suitable for regulating neuronal activity in a limited area such as within a millimeter scale or a deep brain region (4, 5). In contrast, ultrasound is currently attracting great attention as an innovative technology that may change the basic principle of neuromodulation. Although the potential applicability of ultrasound for neuromodulation had already been reported in the 1920 s (6), equipment performance was insufficient for generating adequate irradiation power for transmission through the skull, thereby restricting its brain application during the last decades (7). Nonetheless, recent technical advances in focused acoustic waves have overcome most of the limitations; ultrasound has received renewed interest as a versatile tool for noninvasively controlling brain circuits by excellent tissue penetration and high spatial precision (8, 9).

Despite the expanding utility of ultrasound in the mammalian brain, the mechanisms by which ultrasound alters brain function remain a fundamentally open question. In rodent brains, transcranial ultrasound may directly impact neuronal activity in vivo either by stimulation of targeted tissue (10, 11) or by indirect effects via mechanisms related to the auditory system (12, 13). This means that identification of the key components involved in the mechanism of ultrasound neuromodulation is difficult by relying only on in vivo experimental systems. On the other hand, mouse cortical neurons in dissociated cultures and brain slices lacking any auditory influences also demonstrate robust responses to ultrasound stimulation (14–16). These facts indicate that neurons have an intrinsic ability to directly respond to ultrasound. Given the major bioeffects of ultrasound in living organisms such as mechanical pressure and cavitation (9), these findings indicate that innate mechanosensitive molecules in neurons are critically involved in ultrasound neuromodulation. In fact, mammalian neurons express various types of mechanosensitive ion channels that sense biophysical changes both inside and outside the body (17), and some of them have already been identified as essential players in ultrasound neuromodulation (5). Nevertheless, the experimental evidence provided by previous studies is still mainly controversial and the crucial mechanosensitive channel that contributes to ultrasound neuromodulation has not yet been identified. These issues prompted us to further explore the molecular and cellular bases of the ultrasound-mediated regulation of neuronal functions.

In this research, we established a live-cell imaging assay system based on the application of a fluorescence microscope in combination with an ultrasound stimulator, and therewith we systematically characterized ultrasound-elicited cellular responses in cultured cortical and hippocampal neurons. In parallel, using the same technical paradigm of ultrasound stimulation, we also efficiently validated whether the findings obtained from cell culture-based analysis were consistent with the observation from living mouse brains by in vivo electrophysiological recordings. Notably, our strategy identified transient receptor potential canonical 6 (TRPC6), a mechanosensitive and nonselective cation channel, as a key sensor molecule responsible for the initial biological event of intrinsic neuronal response to ultrasound neuromodulation in mammalian neurons both in vitro and in vivo.

## Results

### Ultrasound Irradiation Induces an Increase of Intracellular Ca^2+^ Concentration in Cultured Neurons.

**Fig. 1.**
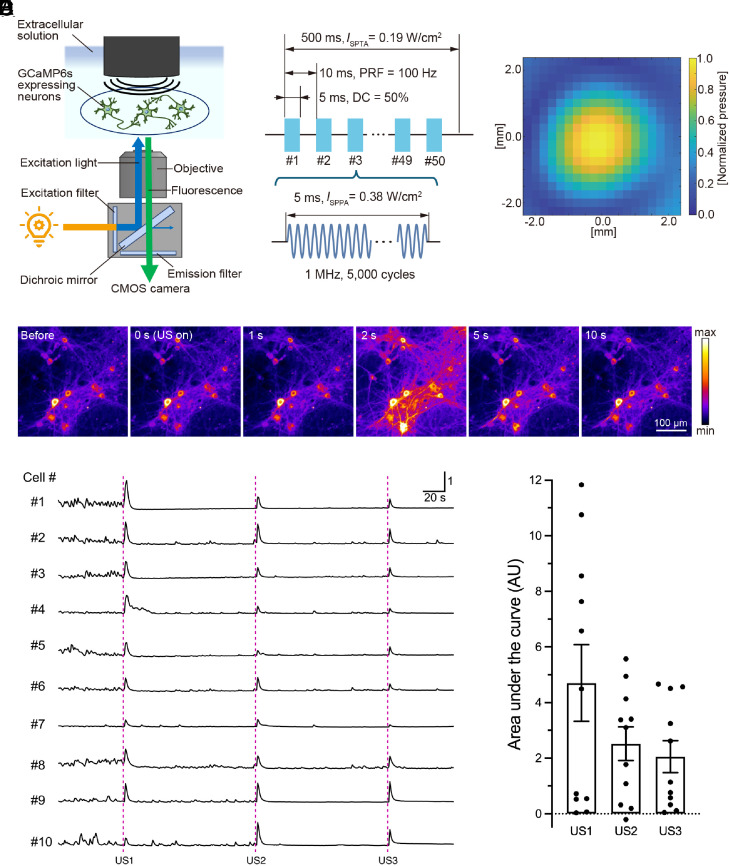
Ultrasound irradiation induces Ca^2+^ transients in cultured cortical neurons. (*A*) A scheme of in vitro experimental setup to monitor ultrasound-induced neuronal response. (*B*) An ultrasound burst pulse sequence used for in vitro experiments. PRF: Pulse repetition frequency, DC: Duty cycle. (*C*) Pressure distribution of stimulating ultrasound measured inside free water. (*D*) Representative sequential images depicting GCaMP6s fluorescence in neuronal cells during pre-, post-, and peristimulation phases induced by ultrasound irradiation. The scale bar demonstrates minimum to maximum values normalized to a global max. (*E*) Representative traces of fluorescence calcium imaging demonstrating pulsed ultrasound irradiation induced rapid Ca^2+^ transients in individual neurons. Ultrasound stimulation at an intensity of 190 mW/cm^2^ and duration of 500 msec is applied to neurons at the time of US1, US2, and US3. (*F*) The amplitudes of Ca^2+^ transients in response to sequential ultrasound stimulation at US1, US2, and US3 are quantified by area under the curve (AU). Bar graph values show mean ± SEM (n = 11, *P* = 0.1349, one-way ANOVA).

To explore the intrinsic mechanisms of neuronal response against ultrasound-mediated neuromodulation, we first established an experimental system that enables the simultaneous monitoring of neuronal activity and controlled stimulation by ultrasound waves. We employed fluorescence calcium imaging to assess intracellular Ca^2+^ dynamics as an optical indicator of cellular activity. Mouse neurons expressing GCaMP6s, a genetically encoded fluorescence calcium indicator, were grown on a coverslip and placed in an imaging chamber perfused with extracellular bath solution on the stage of an inverted widefield fluorescence microscope. In this configuration, the tip of the ultrasound transducer was dipped into the bath solution and set above neurons in a field-of-view, and fluorescence time-lapse imaging was conducted by capturing sequential images with a CMOS camera upward from the bottom side (Fig. 1*A* and *SI Appendix*, Fig. S1). Given the previous studies summarizing the biological applicability of ultrasound-mediated neuromodulation with approximately 0.25 to 2 MHz wave-frequency (18), neurons were stimulated with ultrasound waves at a frequency of 1 MHz. Each stimulus consisted of 100 Hz PRF with a duty cycle of 50% (Fig. 1*B*). A normalized pressure distribution was determined by the measurement of ultrasound in free water using a needle-type hydrophone (Fig. 1*C*).

To establish the appropriate conditions for ultrasound stimulation in which neurons demonstrate a steady response, neurons were sonicated at different stimulation parameters in terms of irradiation intensities (17, 190, 294 mW/cm^2^) and durations (100, 200, 500 msec). At an intensity of 17 mW/cm^2^, neurons barely responded even to a prolonged train of stimulus with a duration of 500 msec. However, ultrasound waves at 190 mW/cm^2^ constantly induced a significant increase in intracellular Ca^2+^ concentration in neurons, and these cellular responses became more robust in a duration-dependent manner (*SI Appendix*, Fig. S2*A*). In contrast, at an intensity of more than 294 mW/cm^2^, ultrasound irradiation frequently caused unstable neuronal responses and relatively large data variability. This instability of the input–output relationship may be due to the unpredictable fluctuation of ultrasound intensity caused by being near the upper limit of the amplifier, or due to the uncontrollable biophysical phenomena caused by potential changes in the shape of the bath solution by the ultrasound radiation force. Therefore, in light of these observations, we chose stimulation parameters at an intensity of 190 mW/cm^2^ and a duration of 500 msec for all subsequent experiments. In this optimized protocol, a rapid increase in the fluorescence intensity of GCaMP6s in neurons was induced within a few seconds after the application of ultrasound stimuli, and these neuronal responses were transient and synchronized among neighboring neurons (Fig. 1*D*). Onset kinetics and the peak amplitude of neuronal responses to ultrasound were also similar to those of neuronal responses elicited by the train of conventional electrical stimuli (*SI Appendix*, Fig. S2*C*). In addition, representative fluorescence traces in independent neurons demonstrated that neurons were repeatedly activated by multiple ultrasound stimulus, with neuronal responses varying in magnitude (Fig. 1*E*). Although the datasets of the respective experimental sessions were relatively variable, the average peak amplitudes in response to three sequential ultrasound stimuli were in the same range and did not differ significantly (Fig. 1*F*).

### Neuronal Responses to Ultrasound Depend on Extracellular Ca^2+^ Influx and Network Excitation.

**Fig. 2.**
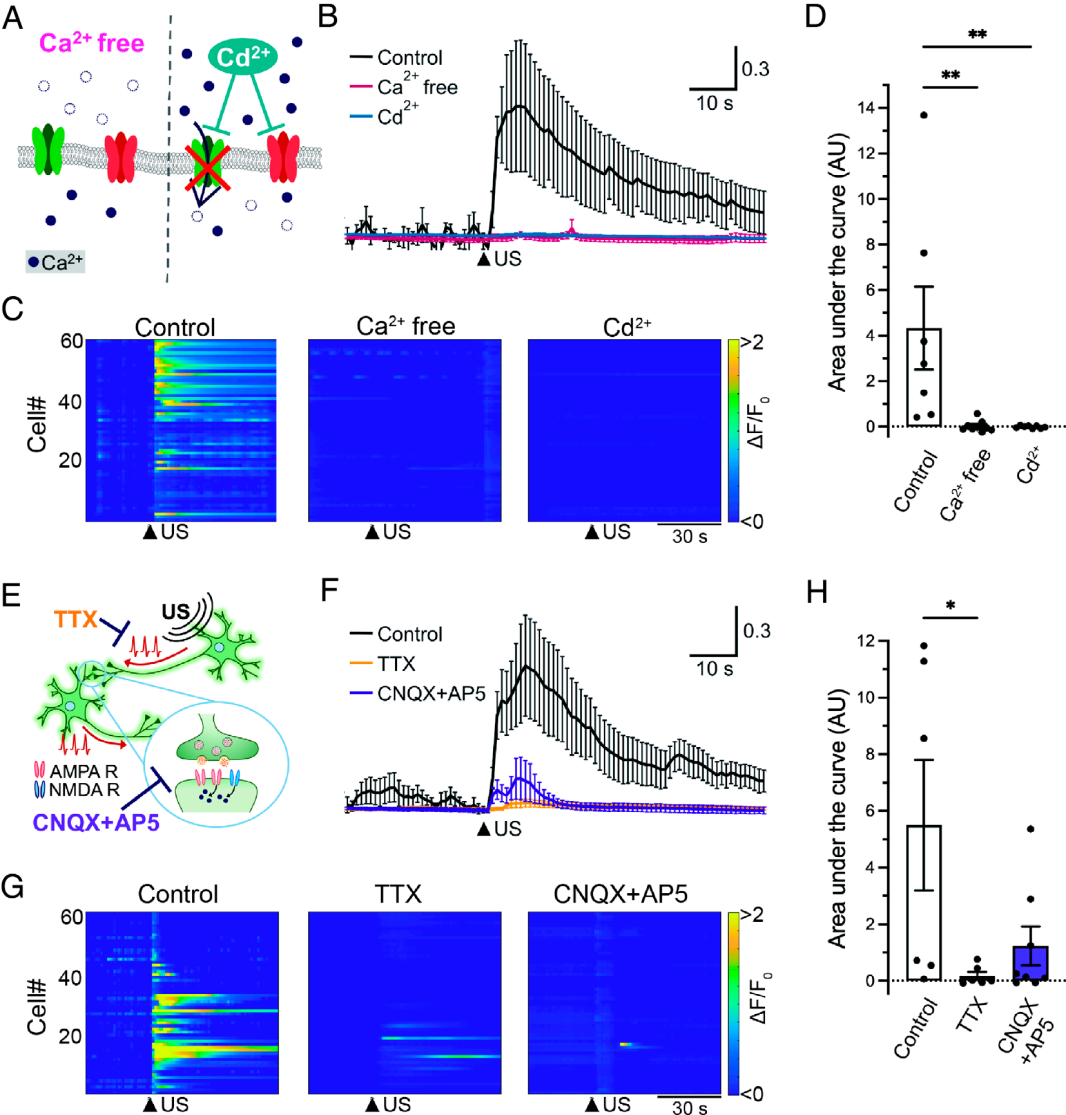
Ultrasound-mediated Ca^2+^ transients depend on extracellular Ca^2+^ influx and network activity. (*A*) A scheme of strategies to investigate the impact of Ca^2+^ influx from outside of the cell on ultrasound-induced neuronal response. Cd^2+^ (100 µM) blocks Ca^2+^-permeable ion channels. (*B*) Averaged traces of neuronal Ca^2+^ transients against ultrasound irradiation under the condition of control (black), Ca^2+^ free (red), or the presence of Cd^2+^(blue). Data from seven, nine, and eight independent experiments for control, Ca^2+^ free, and Cd^2+^ are plotted as mean ± SEM, respectively. The arrowhead indicates the time of ultrasound stimulation. (*C*) Heatmap demonstration of normalized fluorescence intensity (ΔF/F_0_) of GCaMP6s in neurons under the condition of control, Ca^2+^ free, or the presence of Cd^2+^. Data from 60 cells in one experiment are plotted. Arrowheads indicate the time of ultrasound stimulation. (*D*) The amplitudes of Ca^2+^ transients in response to ultrasound stimulation are quantified as the area under the curve (AU). Bar graph values show mean ± SEM. **P < 0.01 (one-way ANOVA followed by the Dunnett post hoc test). (*E*) A scheme of strategies to investigate the impact of the generation of action potentials and functional network activity on ultrasound-induced neuronal response. TTX (1 µM) is used to prevent the generation of action potentials and a mixture of CNQX (10 µM) and AP5 (50 µM) is used to block synaptic transmission. (*F*) Averaged traces of neuronal Ca^2+^ transients against ultrasound irradiation under the condition of control (black), TTX-treated (orange), or CNQX+AP5-treated (purple) neurons. Data from six, six, and eight independent experiments for control, TTX, and CNQX+AP5 were plotted as mean ± SEM, respectively. (*G*) Heatmap analysis of neuronal responses under the condition of control, TTX-treated, or CNQX+AP5-treated neurons. Data from 60 cells in one experiment were plotted. (*H*) Bar graph values show mean ± SEM. *P < 0.05 (one-way ANOVA followed by the Dunnett post hoc test).

Since ultrasound stimuli induced rapid Ca^2+^ transients in neurons within a few seconds, the major source of the increased intracellular Ca^2+^ was assumed to be Ca^2+^ influx from outside of the cell. To address this fundamental question, we first investigated whether Ca^2+^ removal from extracellular bath solution impacts ultrasound-mediated intracellular Ca^2+^ dynamics in neurons (Fig. 2*A*). As expected, we observed that extracellular Ca^2+^-free condition drastically suppressed the Ca^2+^ transients even after the ultrasound stimulation (Fig. 2 *B*–*D*). In addition, ultrasound-mediated neuronal responses were also strongly attenuated in the presence of Cd^2+^, a potent nonselective calcium channel blocker (Fig. 2 *B*–*D*), suggesting that ultrasound-induced neuronal responses rely on extracellular Ca^2+^ influx through calcium channels in the plasma membrane.

As dissociated cortical neurons randomly form interconnections with neighboring neurons and develop spontaneous network activity via excitatory synaptic transmission in culture, next we pharmacologically examined whether ultrasound-mediated Ca^2+^ transients are induced by the enhancement of network activity (Fig. 2*E*). Tetrodotoxin (TTX), a selective blocker of voltage-gated sodium channels (VGSC), inhibited the generation of action potential in individual neurons, and we observed that the toxin treatment elicited no neuronal responses to ultrasound stimuli (Fig. 2 *F*–*H*). We also tested the effect of CNQX and AP5, potent antagonists against AMPA-type and NMDA-type ionotropic glutamate receptors in postsynapse, respectively, on ultrasound-dependent neuronal responses. In the presence of both drug mixtures, neuronal responses to ultrasound were sharply suppressed (Fig. 2 *F*–*H*). Additionally, we confirmed that nifedipine, a selective L-type voltage-gated calcium channel blocker, also significantly reduced ultrasound-induced neuronal responses (*SI Appendix*, Fig. S3). These results suggest that ultrasound-mediated Ca^2+^ transients require the generation of action potentials and functional network activity.

Taken together, our findings indicate that ultrasound-mediated Ca^2+^ transients represent the overall neuronal excitation of network activity arising as a consequence of the biological events involved in ultrasound irradiation.

### Biophysical Effects of Ultrasound on Cultured Neurons.

The biophysical effects of ultrasound on living organisms include temperature rise, cavitation, and acoustic radiation force. To examine the thermal effect of ultrasound, we first performed real-time monitoring of the bath temperature with a thermocouple during ultrasound stimulus at various duty cycles. The temperature rose immediately after sonication and quickly returned to baseline for a few seconds, and the degree of temperature rise correlated well with the % value of the duty cycle (*SI Appendix*, Fig. S4*A*). Nevertheless, ΔTemp_(peak-baseline)_ was less than 0.8 °C even at the maximum peak when ultrasound with a duty cycle of 50% was applied (*SI Appendix*, Fig. S4*B*), and this would be still an overestimated value as viscous heating becomes sources of artifacts in experiments to measure ultrasonic heating. In calculation, the energy of one-shot ultrasound stimulation (0.19 W/cm^2^ × 0.28 cm^2^ × 0.50 s = 0.027 J) causes 0.0064 °C temperature rise of a 1-mL bath solution. Importantly, we also confirmed that a stepwise increase of bath temperature by 2 °C did not change the spontaneous Ca^2+^ oscillation of neuronal culture. (*SI Appendix*, Fig. S4*C*). These data exclude the possibility that ultrasound-induced neuronal responses are caused by mechanisms related to a temperature increase.

Then, the ultrasound waves at a frequency of 1 MHz and an intensity of 190 mW/cm^2^, which we used in all experiments, were below the cavitation threshold (19). To test this idea experimentally, cavitation-mediated free radical generated by ultrasound was measured using a starch-iodine method (20) that detects cavitation generation as a change in the absorbance of the starch solution. The result showed that the absorbance increased with the ultrasound exposure time and saturated at 30 min. No significant difference in absorbance was found between the nontreated and 500 msec exposed conditions, suggesting absent or very little cavitation activity (*SI Appendix*, Fig. S5*A*). Besides, ultrasound stimuli consistently induced similar levels of neuronal Ca^2+^ transient in both standard and degassed bath solutions (*SI Appendix*, Fig. S5 *B* and *C*). Taken together, these results indicate that cavitation is likely not involved in the mechanism of ultrasound-induced neuronal responses.

Based on these results, we hypothesized that acoustic radiation pressure generates a mechanical force to activate mechanosensitive channels in neurons, which may be an initial trigger for neuronal depolarization and further excitation of network activity.

### TRPC6 is an Essential Channel for Ultrasound-Induced Neuronal Responses.

**Fig. 3.**
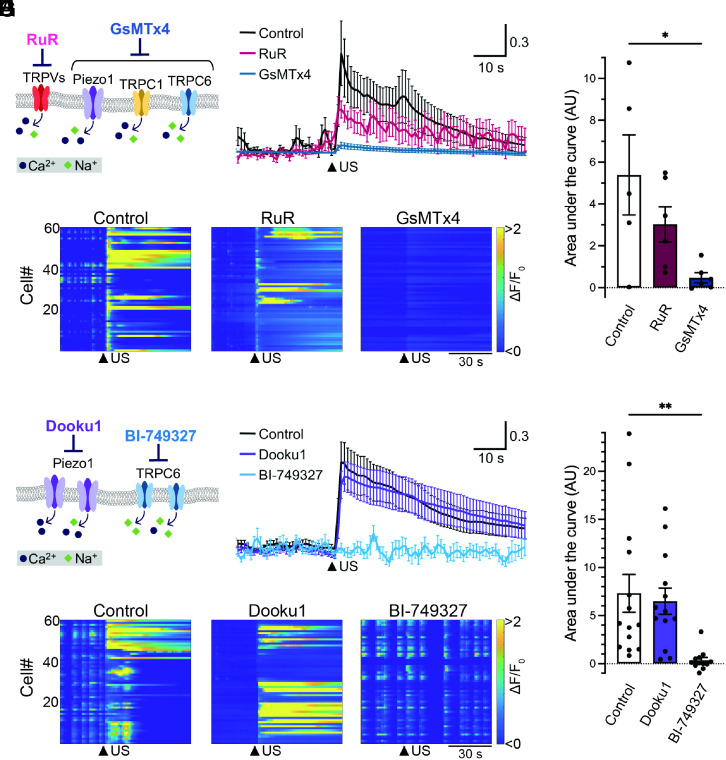
Pharmacological investigation of the involvement of mechanosensitive channels in neuronal responses to ultrasound stimulation. (*A*) A schematic diagram of mechanosensitive receptors and effects of antagonists. RuR (RuR, 5 µM) was used to block TRPVs channel and GsMT×4 (10 µM) was used to prevent gating Piezo1/2, TRPC1, and TRPC6. (*B*) Averaged traces of neuronal Ca^2+^ transients against ultrasound irradiation under the condition of control (black), RuR-treated (red), or GsMT×4-treated (blue) neurons. Data from five, six, and six independent experiments for control, RuR, and GsMTx4 are described as mean ± SEM, respectively. The arrowhead indicates the time of ultrasound stimulation. (*C*) Heatmap demonstration of normalized fluorescence intensity (ΔF/F_0_) of GCaMP6s in neurons under the condition of control, or the presence of RuR or GsMT×4. Arrowheads indicate the time of ultrasound stimulation. Data from 60 cells in an experiment are plotted. Arrowheads indicate the time of ultrasound stimulation. (*D*) The amplitude of Ca^2+^ transients in responses to ultrasound stimulation is quantified as area under the curve (AU). Bar graph values show mean ± SEM. *P < 0.05 (one-way ANOVA followed by the Dunnett post hoc test). (*E*) A scheme of the effects of selective antagonists on Piezo1 or TRPC6. Dooku1 (10 µM) and BI-749327 (1 µM) were used to inhibit Piezo1 and TRPC6 activation, respectively. (*F*) Averaged traces of neuronal Ca^2+^ transients against ultrasound irradiation under the condition of control (black), or the presence of Dooku1 (purple) or BI-749327 (light blue). Fourteen, thirteen, and twelve independent experiments for control, Dooku1, and BI-749327, respectively, were performed. Traces show mean ± SEM. (*G*) Heatmap analysis of neuronal responses under the condition of control, Dooku1 treated, or BI-749327 treated neurons. (*H*) Bar graph values of area under the curve in each condition show mean ± SEM. **P < 0.01 (one-way ANOVA followed by the Dunnett post hoc test).

Since cortical and hippocampal neurons express several potential mechanosensitive channels including TRPV1, TRPV2, TRPV4, Piezo1, TRPC1, and TRPC6 in the mammalian brain (17, 21, 22), we next sought to identify the specific channels that predominantly contribute to ultrasound-mediated Ca^2+^ transients using pharmacological blockers (Fig. 3 *A* and *E*). To narrow down the candidate molecules, we examined the effect of ruthenium red (RuR), the antagonist of the broad spectrum of TRPV channels (Fig. 3 *B*–*D*). In the presence of RuR, neuronal responses to ultrasound tended to be suppressed, but they did not significantly differ between the control group and drug-treated group. We also tested the effect of GsMT×4, the peptide inhibitor of mechanosensitive ion channels including Piezo1, TRPC1, and TRPC6, on the ultrasound responses of neurons. Remarkably, neurons incubated with GsMT×4 strongly reduced ultrasound-evoked responses, suggesting Piezo1, TRPC1, and TRPC6 as potential candidates for an ultrasound-responsible molecule (Fig. 3 *B*–*D*). Thus, we pharmacologically segregated these channel pathways utilizing two additional chemicals BI-749327, a selective TRPC6 antagonist, and Dooku1, a Piezo1 antagonist, on ultrasound-induced neuronal responses. To our surprise, ultrasound-mediated Ca^2+^ transients in neurons had almost completely disappeared in the presence of BI-749327 (Fig. 3 *F*–*H*). In contrast, in our experimental condition, we observed that Dooku1 treatment did not have a significant impact on neuronal responses to ultrasound (Fig. 3 *F*–*H*).

In good agreement with the previous study characterizing that Dooku1 blocks Yoda1-induced Piezo1 activation (23), Yoda1-induced calcium influx in N2a cells was drastically suppressed in the presence of Dooku1, an effect that was recovered by the washout of the drug, supporting the effectiveness and selectivity of Dooku1 (*SI Appendix*, Fig. S6 *A* and *B*). In contrast, although the washout of BI-749327 significantly restored the neuronal responsiveness to ultrasound, neurons still constantly demonstrated Ca^2+^ transients in response to ultrasound stimulus before and even after the washout of Dooku1 as well as neurons in the control condition (*SI Appendix*, Fig. S6 *C* and *D*). These results strongly support the pharmacological activities of both Dooku1 and BI-749327, and exclude potential cell toxicity caused by the drug treatment. We also observed neuronal responses induced by the bath administration of Hyp9, a selective TRPC6 agonist, thus confirming the expression of functional TRPC6 channels in cultured cortical neurons (*SI Appendix*, Fig. S7). These findings indicate that the TRPC6 channel is essential for ultrasound neuromodulation in cultured mouse neurons.

**Fig. 4.**
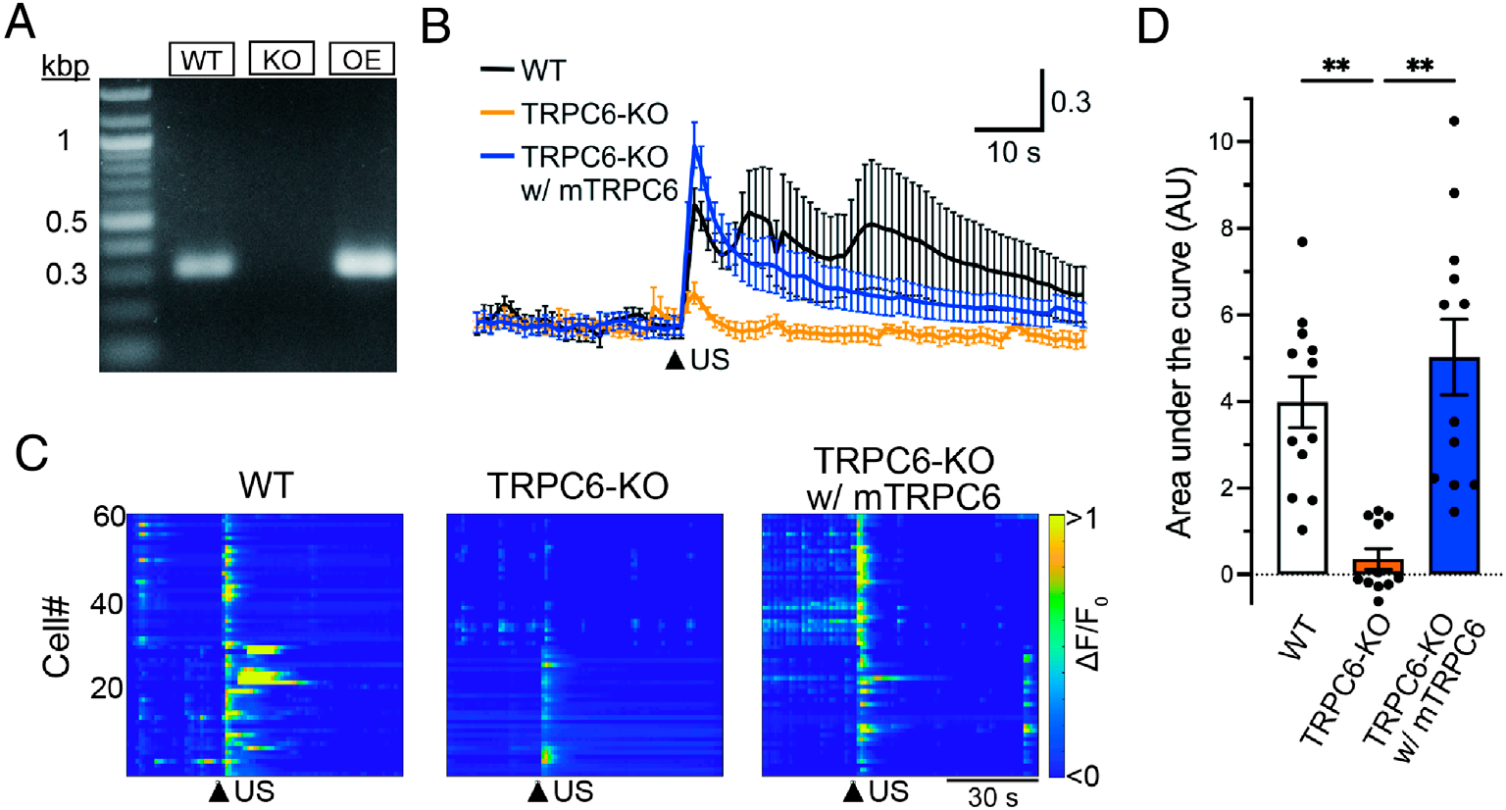
TRPC6-KO neurons lack the response to ultrasound irradiation that can be rescued by overexpression of TRPC6. (*A*) RT-PCR validation of TRPC6 mRNA expression in total RNA extracted from cultured neurons of wild-type (WT), TRPC6 deficient (KO), and TRPC6-KO overexpressing mouse TRPC6-HA (OE) mice. Representative gel electrophoresis images from 2 samples each shows PCR products targeting TRPC6 (327 bp). (*B*) Averaged neuronal responses to ultrasound irradiation of WT (black), TRPC6-KO (orange), or TRPC6-KO overexpressing mouse TRPC6-HA (TRPC6-KO w/ mTRPC6) (blue) neurons. Twelve, eleven, and twelve independent experiments for WT, TRPC6-KO, and TRPC6-KO w/ mTRPC6 neurons, respectively, were performed. The arrowhead indicates the time of ultrasound stimulation. Traces show mean ± SEM. (*C*) Heatmap demonstration of normalized fluorescence intensity (ΔF/F_0_) of GCaMP6s in WT, TRPC6-KO, and TRPC6-KO w/ mTRPC6. Arrowheads indicate the time of ultrasound stimulation. Data from 60 cells in two experiments are plotted. Arrowheads indicate the time of ultrasound stimulation. (*D*) Bar graph values of area under the curve in each condition show mean ± SEM. **P < 0.01 (one-way ANOVA followed by the Dunnett post hoc test).

To further support our findings by genetic experimental evidence, we performed calcium imaging of cultured cortical neurons isolated from the brain of TRPC6-deficient (TRPC6-KO) mouse embryos (Fig. 4*A*). Consistent with the results obtained from the pharmacological experiments, TRPC6-KO neurons showed almost no responses to ultrasound irradiation (Fig. 4 *B*-*D*). Importantly, lentiviral-mediated overexpression of the mouse TRPC6 channel in TRPC6-KO neurons restored the neuronal responsive ability against ultrasound stimulation. RT-PCR validation of TRPC6 expression revealed relatively moderate expression of exogenous mouse TRPC6 transduced by the optimized virus titer in the present study (Fig. 4*A*). Taken together, these results strongly indicate that TRPC6 is a mechanosensitive ion channel directly activated via ultrasound-driven mechanical force in cultured neurons.

### TRPC6 is Essential for Ultrasound Neuromodulation in Mouse Brain.

In order to verify the expression pattern of endogenous TRPC6 in mouse brains, we performed a qPCR-based assessment of TRPC6 expression in brain tissues divided into three regions including the neocortex, hippocampus, and cerebellum. Corresponding to the data resources disclosed by the Allen Brain Atlas (https://mouse.brain-map.org/gene/show/21825), mRNA levels of TRPC6 were significantly more abundant in the neocortex and hippocampus than in the cerebellum, and those expressions were diminished in TRPC6-KO mouse brain (*SI Appendix*, Fig. S8). These findings further indicate the reasonability of our strategy to analyze the ultrasound neuromodulation of neurons derived from the mouse forebrain.

**Fig. 5.**
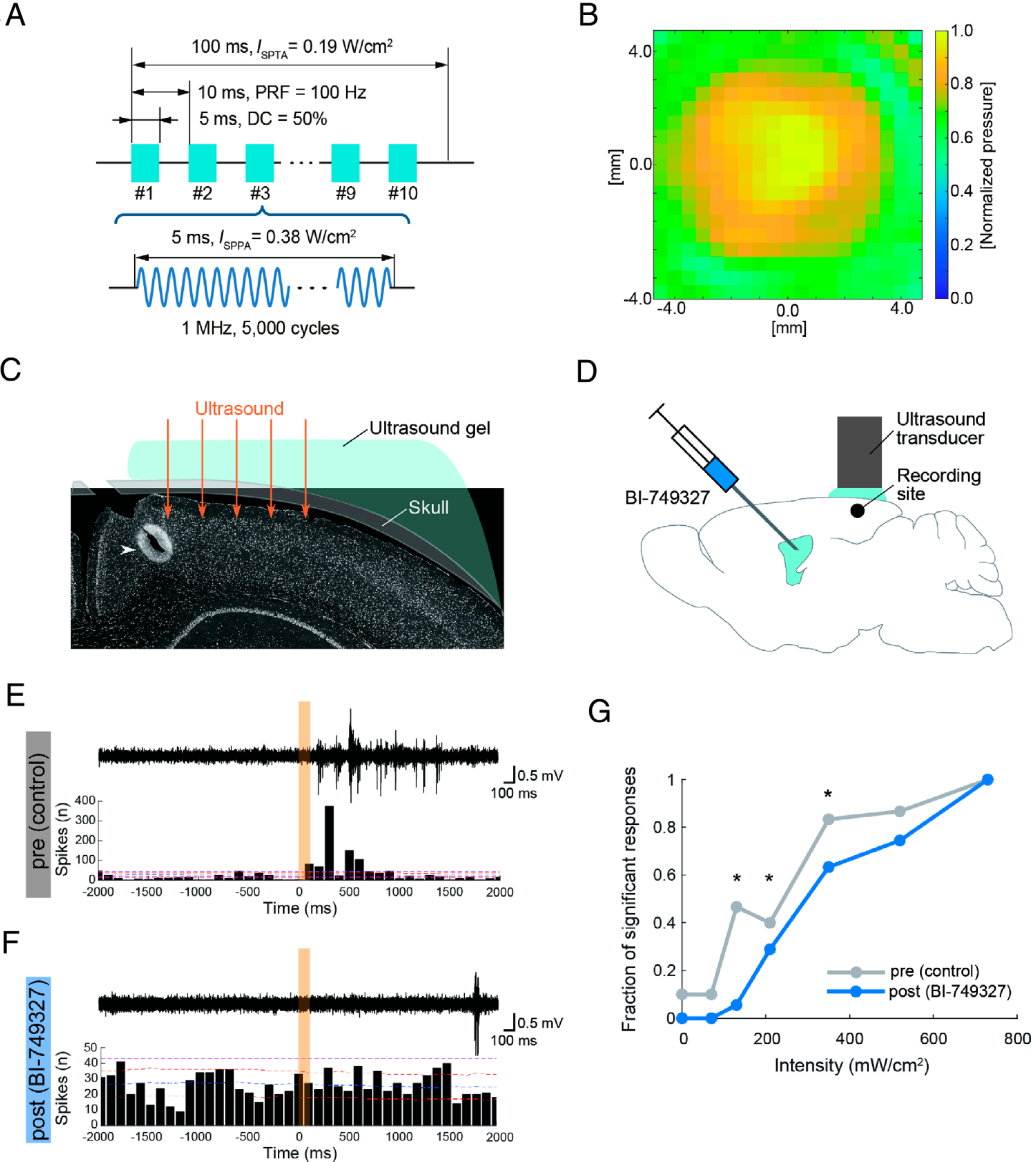
TRPC6 mediates ultrasound-induced neuronal activation in vivo. (*A*) An ultrasound burst pulse sequence used for in vivo experiments. (*B*) Pressure distribution of stimulating ultrasound measured inside free water. (*C* and *D*) Schematic diagrams of in vivo multiunit recordings with a TRPC6 blocker, BI-749327. (*C*) Ultrasound was transcranially irradiated to the cerebral cortex of mice, and a tungsten microelectrode for recordings was inserted through a cranial window. A white arrowhead indicates a representative recording site labeled with an electrical lesion after recordings. (*D*) BI-749327 was intracerebroventricularly administered 30 min before post recordings. (*E* and *F*) Representative in vivo multiunit recordings around US irradiations before (*E*) and after (*F*) BI-749327 administration. *Top*: high-pass filtered extracellular voltage traces; *Bottom*: peristimulus time histograms (PSTHs). Orange patches represent ultrasound irradiation. Dashed pink, red, and blue lines in each PSTH represent global significance levels, local significance levels, and mean counts calculated with 1,000 randomly generated datasets of timestamps of population neural activities with time jittering, respectively. The significance of each recording was determined by whether PSTH goes above or down across the global significant levels. (*G*) Fraction of significantly activated population responses by US irradiations of several intensities. Significance on each stimulus intensity was determined by the Χ^2^ test on a two-by-two (significance × control or BI-749327) frequency table. n = 73, 80 sessions from three mice for pre- and postrecordings, respectively. *P < 0.05.

Finally, to investigate whether TRPC6 has a critical role in ultrasound-induced neuromodulation in the brain, we performed in vivo electrophysiological recordings of neural population activities in the cerebral cortex of anesthetized mice via a tungsten microelectrode with a pharmacological intervention of TRPC6 (Fig. 5 *C* and *D*). Transcranial ultrasound irradiation of 1 MHz in 100 Hz PRF and 50% duty cycle, the same parameter as in our in vitro experiments (Fig. 5 *A* and *B*), significantly induced neural population activities in the cerebral cortex in an intensity-dependent manner (Fig. 5 *E* and *G*; *P* < 0.0001, Χ^2^ test). Notably, consistent with the results of the in vitro experiments, the intracerebroventricular administration of the TRPC6 antagonist BI-749327 significantly reduced activation of cortical neurons induced by transcranial ultrasound irradiation at the intensities of 130, 210, and 350 mW/cm^2^ (Fig. 5 *F* and *G*). The putative TRPC6-mediated ultrasound neuromodulation in vivo was confirmed with a 15 μm-thick silicon probe (*SI Appendix*, Fig. S9 *A* and *B*), which was proved to be free of artifacts originating from possible vibration of the recording electrode by ultrasound irradiation (24). The TRPC6-mediated ultrasound neuromodulation in vivo was also confirmed in chemically deafened mice to exclude the possibility of auditory confounding via the periphery (*SI Appendix*, Fig. S9 *C* and *D*). These results indicate that TRPC6 is a crucial molecule, consistently, in ultrasound neuromodulation both in vitro and in vivo.

## Discussion

In the present study, we established experimental systems to assess neuronal activities evoked by ultrasound irradiation compatible with both cortical neuronal culture and living mouse brain, and we examined the detailed molecular and cellular bases of ultrasound neuromodulation in mammalian neurons. A line of evidence has indicated that the acoustic pressure of ultrasound mechanically impacts neurons, leading to rapid network excitation via the generation of action potentials and synaptic transmission. Most importantly, we successfully identified TRPC6, a mechanosensitive cation channel, as a pivotal biosensor that can detect the mechanical pressure of ultrasound and trigger the sequential biological events of intrinsic neuronal responses. Since ultrasound neuromodulation has attracted increasing attention as an innovative technology to control brain circuits with high spatiotemporal precision, our findings offer crucial insight into the future feasibility of safe and fine-tunable ultrasound neuromodulation supported by scientific evidence, which may eventually contribute to a wide range of neuroscience research fields and disease therapies in humans.

The biophysical impact of ultrasound on living organisms is currently classified into three major categories in the present study, namely, temperature elevation, cavitation, and acoustic radiation force. Temperature elevation is a well-defined characteristic caused by the absorption of ultrasound energy in the targeted medium, and high-intensity ultrasound has been utilized for thermal therapy of brain tumors and movement disorders. As shown in *SI Appendix*, Fig. S4*A*, the temperature rise caused by ultrasound irradiation in our experimental system was both negligible and short-lived in our experimental condition. Besides, as the temperature rise measured by the thermocouple probe we used in this study is frequently overestimated due to viscous heating (25), the actual temperature rise caused by ultrasound is assumed to be much lower. Thus, we believe that the thermal effect is not likely to contribute to the mechanism of ultrasound neuromodulation. As for the issue of cavitation, we consider that it would also be a relatively minor factor for the following reasons. First, on the basis of previous studies, ultrasound at an intensity of 190 mW/cm^2^ is expected to be below the threshold for cavitation generation (19). Second, when we assessed cavitation-mediated free radical generation measured by the starch-iodine method (20), we could not detect a significant difference between the nontreated and sonicated conditions (*SI Appendix*, Fig. S5*A*). Third, ultrasound stimuli consistently induced similar levels of neuronal Ca^2+^ transient in both standard and degassed bath solutions (*SI Appendix*, Fig. S5*B*), and this neuronal response was pharmacologically diminished by several channel blockers represented by Cd^2+^. Fourth, we could not microscopically observe any visible bubbles attached to the neurons that might substantially cause cell damage and toxicity. All evidence in the present study indicates a negligibly small contribution of cavitation to ultrasound neuromodulation of neurons.

TRPC6 is one of the nonselective cation channels widely expressed in various organs including the heart, kidney, and brain (22, 26, 27), and it is involved in the regulation of vascular smooth muscle contractility and arterial blood pressure (28). In agreement with previous studies characterizing neuronal expression and dendritic localization of TRPC6 in mouse forebrain (21, 29, 30) and data resources from the Allen Brain Atlas (https://mouse.brain-map.org/gene/show/21825), we distinctly detected endogenous TRPC6 mRNA in mouse forebrain and cultured cortical neurons. While several research groups have demonstrated that TRPC6 acts as a mechanosensor in cardiomyocytes (31) and podocytes (32) to sense hypoosmotic stretch or indentation of the plasma membrane, there have been no such reports on neuronal cells of the central nervous system. Therefore, the present study provides important evidence that TRPC6 is an intrinsic mechanosensitive channel in cortical neurons essential for ultrasound neuromodulation both in vitro and in vivo.

**Fig. 6.**
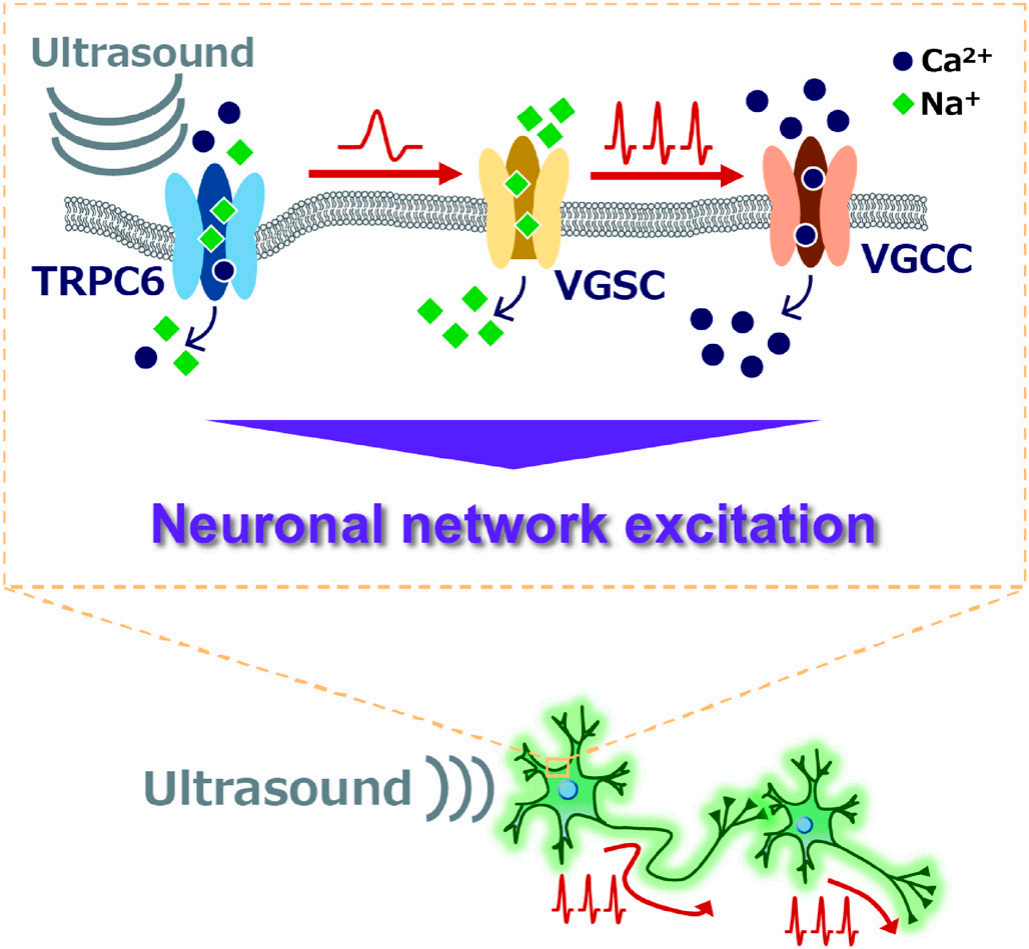
A hypothetical model for the mechanism of ultrasound neuromodulation via TRPC6 activation proposed in the present study. Ultrasound irradiation potentially distorts the cell membrane by acoustic pressure, which triggers the opening of a mechanosensitive TRPC6 channel followed by membrane depolarization in cultured cortical neurons. The generation of action potentials may be induced by the opening of VGSC following the TRPC6-mediated depolarization, which may further elicit extracellular Ca^2+^ influx via voltage-gated calcium channels, resulting in excitation of the neuronal network.

We propose a hypothetical model in which ultrasound-induced Ca^2+^ transient in neurons arises as a consequence of the generation of action potentials and neuronal network activity triggered by TRPC6 activation (Fig. 6). In this model, TRPC6 may act as a biosensor to detect the mechanical distortion of the lipid bilayer structure on the plasma membrane caused by the acoustic pressure of ultrasound. Since spontaneous Ca^2+^ oscillation of neuronal culture was relatively intact in the presence of selective TRPC6 antagonist or the genetic deficiency of TRPC6 (Figs. 3 *G* and 4 *C*), we assume that TRPC6 may not have an essential role in spontaneous network activity. In addition, as TRPC6 has lower calcium ion permeability than other TRP channels such as TRPVs (33, 34), the opening of the TRPC6 channel would preferentially contribute to initial membrane depolarization through sodium influx followed by generation of the action potential in the small neuronal population. Consequently, network excitation with robust Ca^2+^ transient can be amplified through excitatory glutamatergic synaptic transmission (Fig. 6). This sequential biological process presumably takes a couple of hundred milliseconds, and it can explain the delayed onset of neuronal response after ultrasound stimulation (Fig. 5). On the other hand, as we also realize that some neurons demonstrate slightly delayed activation after ultrasound stimuli, this asynchronized response may be involved in the secondary burst excitation of the neuronal network leading to an apparent slow decay of the averaged trace among the entire cell population. Alternatively, a sustained level of intracellular Ca^2+^ concentration may be attributed to the long-lasting modification of the intracellular signaling cascade activated through the downstream process of mechanotransduction. Part of this concept is still hypothetical and further experimental validation will be required to uncover the overall picture of the mechanism.

In vitro culture assay has superior compatibility with pharmacology and genetics, and serves as a powerful experimental platform for screening essential factors involved in ultrasound neuromodulation. Several research groups have independently reported that multiple mechanosensitive ion channels such as TRPC1, TRPP1/2, TRPM4 (16), Piezo1 (14), and N-methyl-D-aspartate receptor (35) are essential in neuronal responses to ultrasound irradiation in mammalian cortical and hippocampal neurons. We consider that these controversial outcomes from several laboratories including from our group may be due to the huge variability among the experimental configurations of the respective laboratories. For instance, while neurons in our experimental system uniformly received direct ultrasound stimulation from above, other groups stimulated neurons by indirect sonication through thin film or glass coverslips from the bottom side of the recording chamber (14, 16). In addition, multiple parameters of acoustic waves (i.e. flat or focused, frequency, intensity, duty cycle, duration, radiation angle, etc) complicate the major bioeffect of ultrasound that may impact the specificity and efficacy of the activation or inactivation of target molecules in neurons (9). In the present study, both genetic and pharmacological evidence strongly indicate that TRPC6 is an essential channel for ultrasound neuromodulation in mouse cortical neurons. Notably, while the specificity of Dooku1 in different states of the Piezo1 channel is still controversial (23, 36, 37), we found that Dooku1 did not have a significant effect on ultrasound-mediated Ca^2+^ transients, implicating that the constitutive activation of Piezo1, if any, induced by acoustic force is not predominantly involved in the biological mechanism with the current condition (Fig. 3). Nevertheless, our study does not exclude the possibility that other mechanosensitive molecules still contribute to ultrasound neuromodulation. Although we have not tested neuronal response elicited by another type of transducer optimized by different ultrasound parameters, there may be multimodal cellular mechanisms that can be triggered by specific frequencies or stimulation patterns of ultrasound (18, 38) as various activation modes of mechanosensitive channels and other membrane proteins have been actively debated (39, 40). In this regard, standardization of experimental systems with optimized ultrasound parameters would be necessary to establish sufficient consensus and comprehensive knowledge for ultrasound neuromodulation.

Another important aspect of our findings is that inhibition of the TRPC6 channel consistently diminishes the neuronal response to ultrasound both in vitro and in vivo. Although immortalized cell lines are relatively easy to handle and maintain, they cannot develop network activity with synaptic connections and would not be an appropriate model for investigating brain function. In contrast, our primary neuronal culture demonstrates spontaneous network oscillation mediated by synaptic transmission and well represents the physiological brain state of living animals. We could reproduce reliable results indicating that TRPC6 is consistently essential for ultrasound neuromodulation both in vitro and in vivo, and therefore it is very likely that our findings reflect the biological phenomena observed in the human brain in clinical studies. We could successfully demonstrate that in vivo ultrasound-induced neuronal activation was not artifact via electrode vibration or auditory confounding via the periphery. Nonetheless, we cannot rule out the possible involvement of other molecules, the biophysical properties of the plasma membrane, and cellular and network mechanisms. For example, we still observed a high probability of neuronal responses to ultrasound even in the presence of TRPC6 blocker when increasing the irradiation intensity (Fig. 5). This may be due to other mechanisms such as reorganization of organelles and cytoskeletons or contribution of other mechanosensitive channels (15, 41). Since diverse cell types such as neuronal subtypes (24), astrocytes (41), and microglia (42) may respond to ultrasound differently, further investigation would be required to fully understand ultrasound neuromodulation.

Current technical advances in a variety of neuromodulation modalities have contributed tremendously to the progress of neuroscience. The most widely used neuromodulation techniques are optogenetics and chemogenetics in animal studies. Optogenetics enables accurate control of neuronal activities by light illumination with high spatiotemporal resolution, but invasive surgical implantation of optical fiber is required. In contrast, chemogenetics achieves robust alteration of basal neuronal activities by systemic drug administration in a less invasive manner, although chemogenetics lacks spatial or temporal resolution, which are both important for selective and effective intervention of the neuronal network in vivo (43). Importantly, both optogenetics and chemogenetics require gene transduction in targeted brain circuits by local virus injection or DNA electroporation, and therefore these approaches are not easily applicable to the human brain. Compared to these technologies, ultrasound has superior advantages in terms of its noninvasiveness and remarkable tissue permeability. In addition, the transcranial ultrasound wave can be focused to concentrate its acoustic energy in the target region, offering unique applications to modifying specific brain circuits with high spatiotemporal regulation. Indeed, a recent study has demonstrated that transcranial focused ultrasound stimulates the hypothalamus preoptic area via TRPM2 channels and causes torpor-like hypothermic and hypometabolic states in rodents (44), promising the feasibility of ultrasound neuromodulation of deep brain circuits in living animals. Furthermore, growing evidence has highlighted the conceptual advance of sonogenetics, and an approach to utilizing genetically encoded ultrasound-responsive mechanosensitive molecules for control of neural activity of genetically defined neuronal population, as a next-generation neuromodulation technology (9, 18, 45). Although the applicability of multiple mechanosensitive channels, including the mechanosensitive channel of large conductance (MscL, a thoroughly analyzed force sensor of *Escherichia coli*) (46–48), Piezo1 (49) and TRPA1 (41, 50) for sonogenetics has been reported in several laboratories, it is still in its early phase and the topic is beyond the scope of the present study. Additional effort would be necessary in order to establish sonogenetics as a mature technical platform in neuroscience.

In conclusion, we found that TRPC6 is an essential intrinsic mediator of ultrasound neuromodulation in cortical neurons of the mammalian brain. Since it has been reported that transcranial ultrasound neuromodulation ameliorates brain pathologies and behavior abnormalities of animal models for neurodegenerative disorders, such as Parkinson’s Disease and Alzheimer’s Disease (51, 52), TRPC6 may contribute to the beneficial mechanism in ultrasound neuromodulation and can also be an attractive pharmacological target for disease-modifying therapies. Our findings provide valuable insight for the encouragement of further investment in ultrasound neuromodulation, and the application of this modulatory technology to animal models will provide in vivo evidence for pivotal roles played by the identified mechanical processes.

## Materials and Methods

### Animals.

Animal experiments were performed and reported according to the National Research Council’s Guide for the Care and Use of Laboratory Animals, institutional guidelines of the National Institutes for Quantum Science and Technology and Hokkaido University, and the Animal Research: Reporting in Vivo Experiments (ARRIVE) guidelines. C57BL/6 J mice were purchased from Japan SLC (Hamamatsu, Japan) or Oriental Yeast Co., Ltd. (Itabashi, Tokyo). TRPC6 deficient mouse strain (TRPC6-KO; B6;129S-*Trpc6^tm1Lbi^/*Mmjax, Stock # 37345-JAX) was purchased from the Jackson Laboratory (Bar Harbor, ME) (28). All mice were housed under specific pathogen-free condition and maintained in a 12 h light/dark cycle with ad libitum access to standard diet and water.

### Cultured Neuron Preparation In Vitro.

Primary neuronal culture was prepared as described previously (53). Briefly, the neocortex and hippocampus of brain tissues were dissected from either C57BL/6 J or TRPC6-KO mice at embryonic days 16 to 18. Neurons were isolated by digesting the collected tissues using papain (Worthington, Lakewood, NJ) in Hank’s Balanced Salt Solution (HBSS, Invitrogen, Waltham, MA). The isolated cells were suspended in Neurobasal-A medium (Invitrogen) supplemented with 10% fetal bovine serum (HyClone, Cytiva, Tokyo, Japan), 1% GlutaMAX (Invitrogen), and 1% N2 supplement (Invitrogen), and plated onto Poly-D-Lysine coated 12 mm glass coverslips (Corning, NY) at a density of 250,000 to 300,000 cells/cm^2^. The next day (DIV1), the medium was replaced by Neurobasal-A medium supplemented with 2% fetal bovine serum, 1% GlutaMAX, and 2% B27 supplement (Invitrogen). For calcium imaging, neuronal cells at DIV1 were infected with a recombinant adeno-associated virus (AAV) encoding GCaMP6s, a genetically encoded fluorescent calcium indicator, driven by synapsin promoter (Addgene, 100843-AAV9, 2 × 10^9^ vg/coverslip) (54). The neurons were maintained by replacing half of the medium with fresh Neurobasal-A medium supplemented with 1% GlutaMAX and 2% B27 supplement every 3 d and used for experiments at DIV12 to DIV16.

### Ultrasound and Electrical Stimulation In Vitro.

Ultrasound stimulation was conducted using a 6-mm diameter flat transducer with 1-MHz center frequency (ST-TM1-6, NEPA GENE, Chiba, Japan) driven by SonoPore KTAC-4000 (NEPA GENE), an ultrasound amplifier to provide programmed burst pulses. We used a 1-MHz ultrasound burst pulse sequence of 500 msec in duration. The pulse sequence contains 50 burst pulses of 100 Hz in pulse repetition frequency and 50% in the duty cycle as otherwise described (Fig. 1*B*).

Total acoustic power delivered from the tip of the transducer was measured with an ultrasound power meter (UPM-DT-10PA, OHMIC Instruments, St. Charles, MO) according to the manufacturer’s protocol. Briefly, the tip of the transducer was held in degassed water above a conical target, and the total downward force on the target proportional to radiation force was directly measured during ultrasound irradiation. *I*_SPTA_ of the pulse sequence calculated as a total acoustic power divided by a radiation surface area of the transducer was 0.19 W/cm^2^, and *I*_SPPA_ of a 5-msec burst pulse was 0.38 W/cm^2^ (Fig. 1*B*).

To induce electrically evoked neuronal excitation as a reference, the extracellular electric train stimulus composed of 10 pulses (each pulse given at 10 V at a duration of 1 msec) was generated by Master-8 pulse stimulator with ISO-FLEX flexible stimulus isolator (A.M.P.I., Jerusalem, Israel). A tip of tungsten concentric bipolar microelectrode (World Precision Instruments, Sarasota, FL) was positioned 1.5 µm above the neurons.

### Fluorescence Calcium Imaging.

Fluorescence calcium imaging for optical recording of neuronal activity was performed with an inverted widefield fluorescence microscope (IX83, Olympus, Tokyo, Japan) controlled by Olympus Cell Sense Dimension software. Cultured neurons expressing GCaMP6s grown onto a coverslip were transferred to the imaging chamber attached to the stage of the microscope. During each imaging session, neurons were maintained by continuous perfusion with HEPES-buffered bath solution (130 mM NaCl, 5 mM KCl, 10 mM HEPES, 10 mM D-glucose, 2 mM CaCl_2_, 1 mM MgCl_2_) at a rate of 1.5 to 2.0 mL/min, in which neurons received minimum mechanical effect from fluid pressure. For time-series analyses of changes in fluorescence intensity, high-resolution images were captured at 1 Hz by Orca-Flash4.0 CMOS camera (Hamamatsu Photonics, Hamamatsu, Japan) with ×20 objective lens (PlanApo, NA 0.45) at room temperature (22 to 25 °C). At the end of each imaging session, we added 50 mM KCl into the bath solution for depolarizing all neurons to make sure neurons are still alive and in a healthy state (*SI Appendix*, Fig. S2*B*). Acquired image stacks were next analyzed by NIH ImageJ / Fiji software. To quantify the fluorescence signal intensity of GCaMP6s as an intracellular Ca^2+^ concentration in individual neurons, regions of interest (ROI) including soma of single neurons were manually placed in each image. Approximately 30 to 60 ROIs per coverslip were randomly selected to measure the Ca^2+^ transients of each neuron, and the averaged grayscale values of each ROI were extracted for data plotting and quantitative analysis. Baseline fluorescence (F_0_) was calculated as the averaged fluorescence intensities for 30 s during the prestimulation period. Normalized fluorescence value (ΔF/F_0_) was then determined as ΔF/F_0_ = (F(t)-F_0_) / F_0_. For measuring the peak magnitude of Ca^2+^ transient elicited by stimuli, the ΔF/F_0_ values for 10 s during the poststimulation period were summed to calculate the area under the curve. All data analysis and plots were prepared using Igor Pro (WaveMetrics, Portland, OR) and GraphPad Prism (GraphPad Software). The heatmaps of normalized fluorescence intensity (ΔF/F_0_) of GCaMP6s in neurons were generated using MATLAB (MathWorks, Natick, MA).

### Pharmacological In Vitro Experiments.

A Ca^2+^-free solution was prepared by simply omitting CaCl_2_ from the bath solution. Cadmium at 100 µM was used as a nonselective Ca^2+^ ion channel blocker. Tetrodotoxin (TTX; Tocris Bioscience, Bristol, UK) at 1 µM was used to prevent the generation of action potentials. To inhibit synaptic transmission, CNQX (Tocris Bioscience) and AP5 (Tocris Bioscience) were used as selective antagonists against AMPA and NMDA receptors at 10 µM and 50 µM, respectively. Nifedipine (Cayman Chemical, Ann Arbor, MI), RuR (Cayman Chemical), GsMTx4 (PEPTIDE INSTITUTE, Osaka, Japan), BI-749327 (MedChemExpress, Monmouth Junction, NJ), and Dooku1 (MedChemExpress) were used to block L-type voltage-gated calcium channel, TRPVs channels, mechanosensitive ion channels, TRPC6 channel, and Piezo1 at 100 µM, 5 µM, 10 µM, 1 µM, and 10 µM, respectively. All chemicals described above were added to the bath solution and preincubated for 5 min prior to ultrasound irradiation in each experimental session. Yoda1 (Cayman Chemical) and Hyp9 (MedChemExpress) were used to activate Piezo1 and TRPC6 at 5 µM and 10 µM, respectively, and added to the bath solution in an experimental session.

### Ultrasound Stimulation and Electrophysiological Recording In Vivo.

Male, 7 to 21 wk old, 20 to 30 g, C57BL6/J mice were used for all the following in vivo experiments. Mice were anesthetized with 0.5 to 1.5% isoflurane followed by subcutaneous administration of 0.1 to 0.3 mg/kg atropine, and they were mounted on a stereotaxic frame. Ground and reference screw electrodes were fixed on the skull above the cerebellum. A small cranial window (~1 mm in diameter) was made at 2.06 mm posterior from bregma and 0.36 mm rightward from the midline. A tungsten microelectrode (TE0.2-70-B-2; UNIQUE MEDICAL Co., Ltd., Tokyo, Japan) or a 15 μm-thick silicon probe (A1×32-Poly3-10 mm-50-177 or A1×16-Poly2-5 mm-50 s-177, NeuroNexus Technologies, Michigan) was inserted through the cranial window and advanced at a 45° angle toward the right side of the coronal plane. Signals via the microelectrode were fed to a preamplifier (C3324; Intan Technologies, CA), and the amplified signals were recorded by recording controller (C3004, Intan Technologies) at a sampling rate of 20 kHz.

A 3 mm-diameter flat transducer (ST-T1-3, NEPA GENE) was used with SonoPore KTAC-4000 (NEPA GENE). Basic parameters of the ultrasound burst pulse sequence are the same as that used for in vitro studies, but the train contains 10 burst pulses, and the duration is 100 msec (Fig. 5*A*). Fig. 5*B* shows a normalized pressure distribution in the free water measured on the plane parallel to the ultrasound transducer surface at a distance of 3 mm. We scanned 19 ×19 points at an interval of 0.5 mm. The peak pressure amplitude at the distribution center was 160 kPa. Simple rectangular but not smoothed (10) stimulus waveforms were employed. The possibility of auditory confounding via the periphery was eliminated via other experiments in chemically deafened mice (*SI Appendix*, Fig. S9 *C* and *D*). The transducer was placed above the right side of the skull and its tip was acoustically coupled to the skull with an ultrasound gel (LOGIQLEAN, GE HealthCare). At each recording site, several recording sessions at different ultrasound intensities (0, 70, 130, 210, 350, 520, 730 mW/cm^2^) were performed. Ultrasound stimulations were performed 20 times with a random interstimulus interval ranging from 10 to 20 s. After recordings at each site, the electrode was advanced by 50 to 100 µm for the next recording sessions.

For pharmacological intervention of TRPC6, BI-749327 (MedChemExpress) was dissolved with a vehicle composed of 10% DMSO, 40% PEG300, 5% Tween-20, and 45% physiological saline, and stored at 4 °C at a concentration of 5 mM. Before the experiments, the stock solution was further diluted to 0.6 mM with saline and 2 µL of the diluted solution was intracerebroventricularly administered after the control recording sessions using a NANOFIL microsyringe (World Precision Instruments; FL) and ultramicro pomp (UMP3T-1, World Precision Instruments) at a flow rate of 10 nL/s. Intracerebro-ventricular administration rather than local cortical microinjection was employed so as not to induce much shear stress to the recording site in the cerebral cortex. Recordings were resumed 30 min after administration.

After the data acquisition, 100 µA anodal direct currents were passed for 10 s via the electrode tip for the electrical lesion to locate the recording sites. The mice were then transcardially perfused with physiological saline followed by 4% PFA and 0.2% picric acid in 0.1 M PB. After post fixation and cryoblockade with sucrose infiltration, 50 µm-thick coronal brain sections were prepared with a cryostat. The sections were stained with 1 µg/mL DAPI in 0.1 M PB for 15 min, coverslipped, and observed by confocal microscopy (LSM900, Carl Zeiss, Oberkochen, Germany).

### Detection of Ultrasound-modulated Population Neural Activity In Vivo.

For the detection of modulated neural activity by ultrasound irradiation, PSTH were prepared for each recording session, and cross-correlation (CCG) analysis was conducted between timestamps of stimulus and population (multiunit) neural activities with MATLAB (MathWorks, Natick, MA). Randomly time-jittered 1,000 datasets of timestamps of population neural activities were generated by adding random time jitters ranging from −3 s to +3 s to the real timestamps of the population neural activities. Pointwise 95% acceptance bands were calculated from the jittered datasets for each 100 msec bin. Multiple comparison error was corrected by introducing “global significance bands” (55). The ultrasound-induced modulation of population neural activities was considered to be significant if any of its CCG bins went across the global significance bands within 1,000 msec after the ultrasound irradiation.

### Statistics.

Statistical analysis was performed by either GraphPad Prism or Microsoft Excel software. Unpaired Student’s *t* test and one-way ANOVA followed by Dunnett’s multiple comparisons test were employed for comparisons between two-group data and three- or more group data of in vitro experiments, respectively. Χ^2^ test for independence was employed to test the data of fractions of significantly modulated population neural activities of the in vivo experiment.

## Supplementary Material

Appendix 01 (PDF)

Dataset S01 (XLSX)

## Data Availability

All of the data underlying the figures are available at Zenodo (https://doi.org/10.5281/zenodo.14004997) (56).
