# Supplementary material for: TRPC6 is a mechanosensitive channel essential for ultrasound neuromodulation in the mammalian brain
Source: Proc Natl Acad Sci U S A. 2024 Dec 3;121(50):e2404877121. doi: 10.1073/pnas.2404877121 (PMC11648612; doi:10.1073/pnas.2404877121)
Supplement: Supplementary file 1 — Appendix 01 (PDF) [file pnas.2404877121.sapp.pdf]

PNAS

Supporting Information for

TRPC6 is a mechanosensitive channel essential for ultrasound neuromodulation in mammalian brain

Yumi Matsushita[1, †], Kaede Yoshida[3, †], Miyuki Yoshiya[1], Takahiro Shimizu[1], Satoshi Tsukamoto[2], Nobuki Kudo[4], Yuichi Takeuchi[3, 5, *], Makoto Higuchi[1], Masafumi Shimojo[1, *]

[1]Advanced Neuroimaging Center, National Institutes for Quantum Science and Technology, Chiba 263-8555, Japan
[2]Laboratory Animal and Genome Sciences Section, National Institutes for Quantum Science and Technology, Chiba 263-8555, Japan
[3]Department of Biopharmaceutical Sciences and Pharmacy, Faculty of Pharmaceutical Sciences, Hokkaido University, Sapporo 060-0812, Japan
[4]Laboratory of Biomedical Engineering, Division of Bioengineering and Bioinformatics, Faculty of Information Science and Technology, Hokkaido University, Sapporo 060-0814, Japan

[5]Laboratory of Pharmacotherapy, Department of Pharmacy, Faculty of Pharmacy, Kindai University, Osaka 577-8502, Japan

[†]Y.M., K.Y. contributed equally to this work

* Corresponding authors:

Masafumi Shimojo, Ph.D., Advanced Neuroimaging Center, National Institutes for Quantum Science and Technology, Chiba 263-8555, Japan. Phone: +81-43-206-3249, Email: shimojo.masafumi@qst.go.jp

Yuichi Takeuchi, Ph.D., Laboratory of Pharmacotherapy, Department of Pharmacy, Faculty of Pharmacy, Kindai University, Osaka 577-8502, Japan. Phone: +81-6-4307-3651, Email: ytake@phar.kindai.ac.jp

This PDF file includes:

Materials and Methods
Figures S1 to S9
Reference

Other supporting materials for this manuscript include the following:

Dataset S1

Supplemental methods

Cell culture

Mouse Neuroblastoma Neuro2a (N2a) cells, well-characterized cells natively expressing Piezo1 (1), were cultured in high-glucose DMEM (Invitrogen) supplemented with 10% fetal bovine serum (Sigma) and Penicillin/Streptomycin (Invitrogen) in a 5% CO_2 incubator at 37°C. For calcium imaging, cells were plated onto Poly-D-Lysine coated 12 mm glass coverslips and transfected with plasmid DNAs encoding GCaMP6s driven by CMV promoter using FuGENE reagent (Promega, WI, USA) following the manufacturer's protocol. 24 hours after transfection, the cells were moved to the imaging chamber for further live-cell calcium imaging.

Pressure distribution and reflection of ultrasound

Fig. 1C shows a normalized pressure distribution in free water measured using a needle-type hydrophone (NH0500, Precision Acoustics, Dorchester, UK) and a laboratory-developed acoustic measurement system (AMS). The peak pressure amplitude at the distribution center was 140 kPa. The hydrophone has a sensor element of 0.5 mm in diameter and senses a pressure area of 0.2 mm^2, calibrating at the frequency range of 1 MHz to 20 MHz. AMS is a computer-controlled XYZ-axis scan and data acquisition system. The spatial resolutions of the scan are 2 μm in an XY scanner (MSS-B1110, Chyuo Precision Industrial, Tokyo, Japan) and 1.6 μm in a Z scanner (SPL28T08-60, Oriental Motor, Tokyo, Japan). Pressure waveforms measured by the hydrophone were digitized by an oscilloscope (TDS2002B, Tektronix, Tokyo, Japan) and transferred to the computer. Scan control, data acquisition, and data processing were performed using LabVIEW (National Instruments, TX, USA). Pressure distribution was acquired on the plane parallel to the ultrasound transducer surface at a distance of 5 mm. We scanned 19 x19 points at an interval of 0.25 mm. Pressure amplitudes were derived as root mean square values of the waveforms.

Fig. S1C shows an exposure chamber used for ultrasound stimulation of the neurons. About 1 mL of bath solution was dropped on the center of a glass-bottom imaging chamber (CK-1, Narishige, Tokyo, Japan), and a 12 mm diameter coverslip with neurons cultured on its surface was slipped into the bath solution. A tip of the transducer was then immersed into the bath solution at a distance of 5 mm above the neurons. Characteristic acoustic impedances of coverslips of the imaging chamber and cell culture (Z_g) were estimated to be 1.2 x 10^7 kg/(m^2s), and that of the medium (Z_m) is 1.5 x 10^6 kg/(m^2s). The reflection coefficient at a medium-glass boundary ($R_{m/g}$)

was 0.78, and those at a medium-air boundary and glass air boundary are almost -1 (Fig. S1D).

Virus preparation for overexpression

For the overexpression of TRPC6 protein, a cDNA fragment encoding mouse TRPC6 (Accession# NM_013838) fused to HA epitope tag was synthesized and subcloned into the multi-cloning site of a lentivirus shuttle plasmid containing human Ubiquitin C promoter. HEK293T cells were maintained in high-glucose DMEM (Invitrogen) supplemented with 10% fetal bovine serum (Sigma) and Penicillin/Streptomycin (Invitrogen) at 37°C in a 5% CO_2 incubator. Recombinant lentivirus packaging was performed in HEK293 cells by co-transfection of lentivirus shuttle plasmid and packaging plasmids pVSVg, pRev, and pGag/Pol using FuGENE reagent (Promega, WI, USA). 48 hours after transfection, lentivirus particles secreted in the culture medium were harvested, purified with a 0.45 μm filter, and stored at -80°C until use. The lentivirus was infected to cultured neurons at DIV5 by direct addition of viral solution into the culture medium (30 μl virus per 1 mL).

RT-PCR and Quantitative real-time PCR

For RT-PCR, total RNA was extracted from cultured neurons using RNeasy Mini Kit (Qiagen, Hilden, German) and purified using RNase-Free DNase Set (Qiagen) according to the manufacturer's instructions. Reverse transcription was performed with 500 ng of total RNA and PrimeScript™ RT Master Mix (Perfect Real Time) (Takara, Tokyo, Japan). Reverse transcription PCR (RT-PCR) was performed with 2 μL cDNA, primers for TRPC6 (from 5' to 3': sense, AAA GAT ATC TTC AAA TTC ATG GTC; antisense, CAC GTC CGC ATC ATC CTC AAT TTC), and Q5 High-Fidelity 2X Master Mix (New England Biolabs, Ipswich, MA, USA). RT-PCR protocol was involved in 35 cycles of PCR at 98°C for 10 sec, at 59°C for 30 sec, and at 72°C for 30 sec in TaKaRa Thermal Cyclar Dice Touch (Takara). For Quantitative real-time PCR (qPCR), total RNA was extracted from the neocortex, hippocampus, and cerebellum of WT or TRPC6- KO mice using RNeasy Lipid Tissue Mini (Qiagen) and purified using RNase-Free DNase Set (Qiagen) according to the manufacturer's instructions. Reverse transcription was performed with 1.5 μg of total RNA and PrimeScript™ RT Master Mix (Perfect Real Time) (Takara, Tokyo, Japan). qPCR was performed with 2 μL cDNA, 2 pairs of primers designed for TRPC6 (from 5' to 3': sense, AAA GAT ATC TTC AAA TTC ATG GTC; antisense, CAC GTC CGC ATC ATC CTC AAT TTC) or beta-actin (from 5' to 3': sense, CAT CCG TAA AGA CCT CTA TGC CAA C; antisense, ATG GAG CCA CCG ATC CAC A), and PowerUp SYBR Green Master Mix for qPCR (Thermo Fisher Scientific, Waltham, MA, USA). qPCR protocol involved 40 cycles of PCR at 95°C for 15 sec and at 60°C for 1 min in Quant Studio 3 (Thermo Fisher Scientific). For each primer pair, the linearity of the reaction was confirmed by the calibration curve with diluted cDNA from the first-strand synthesis

(1, 1/5, and 1/25). The amount of target DNA in the cDNA preparations was quantified by the ΔΔCt method using beta-actin as an internal control.

Assessment of the thermal effect

Ultrasound-mediated thermal effect was measured using a thermocouple probe connected to TC-324C temperature controller (Warner Instruments, Holliston, MA, USA). Briefly, the ultrasound transducer was positioned in the glass-bottom imaging chamber under the same conditions as for fluorescence calcium imaging. A tip of the probe was placed just below the transducer in the bath solution, and the local bath temperature was monitored during sonication with duty cycles at 10%, 20%, 30%, 40%, and 50%. Output data was acquired with PowerLab analog/digital converter system at 1 kHz of sampling rate and analyzed using LabChart software (AD Instruments, Castle Hill, NSW, Australia). To assess the thermal effect on neuronal activity, the temperature of the bath solution was gradually increased by about 0.5°C every 4 minutes using SH-27B In-Line heater (Warner Instruments, Holliston, MA, USA) during calcium imaging of the cultured neurons. The sampling rate of changes in neuronal fluorescence intensity was at 0.2 Hz. Bath temperature in the chamber was measured by a thermocouple probe simultaneously.

Measurement of cavitation-mediated free radical generation

Cavitation-mediated free radical generated by ultrasound irradiation was determined using a starch-iodine method as described previously (2). Briefly, the assay solution composed of 0.01 M KI, 0.1 M CCl_3CHO, 1 M NaCl, and 0.3 mg/mL starch was prepared and sonicated with 1-MHz ultrasound burst pulses for 0.5 seconds, 5 minutes, 15 minutes, 30 minutes, and 1 hour, respectively. In this protocol, H_2O_2 produced by cavitation oxidizes KI to liberate I_2 which further forms a purple-colored complex with starch leading to colorimetric changes in the assay solution. Absorbance at a wavelength of 555 nm corresponding to the stoichiometric ratio of the colored complex was measured using a DS-11 Spectrophotometer (DeNovix Inc., Wilmington, DE, USA). 5% H_2O_2 was also tested as a positive control of the reaction.

Inducing chemically deafened mice

Anesthetized mice received tail vein injection of 175 mg/kg furosemide (079-02973, FUJIFILM Wako Pure Chemical Corporation, Osaka, Japan) followed by subcutaneous injection of 350 mg/kg gentamicin (F4381-1G, Sigma-Aldrich, St. Luis, USA) (3-5). 72 hours later, the mice were tested for hearing loss by responding to 70-80 dBm sound. The mice confirmed as deaf were used in the following electrophysiological recordings with tungsten microelectrodes.

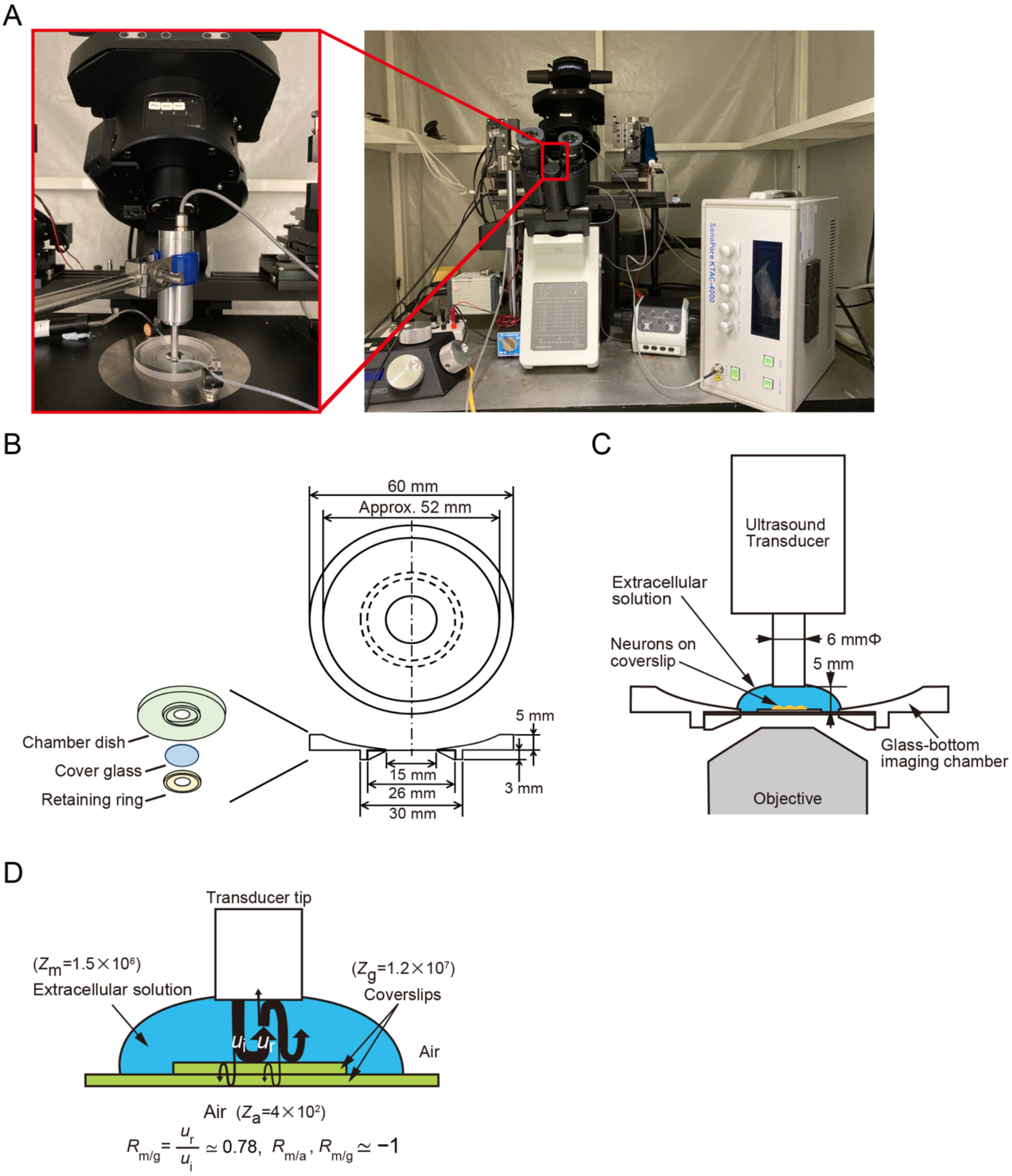

Fig. S1. Experimental configuration of a fluorescence microscope and ultrasound stimulus

A) Actual pictures of the experimental setup and pieces of equipment for *in vitro* calcium imaging connected to an ultrasound transducer. B) Dimensioned drawing of the imaging chamber. C) A setup for *in vitro* ultrasound stimulation under microscopic observation. D) Multiple reflections of ultrasound inside the extracellular solution and coverslips. Since our *in vitro* experiments essentially require real-time microscopic observation of neuron activities under ultrasound irradiation, the experimental setup adopted a glass bottom dish designed to be used with an inverted-type microscope. In this situation, ultrasound initiated from the transducer makes multiple

reflections at the medium boundaries with air and glass, generating a complex standing wave field inside the droplet. Interference of initial incident ultrasound (u_i) with its non-inverting reflection (u_r) at the medium-coverslip boundary makes an amplitude of ultrasound pressure irradiated to the neurons on the coverslip surface 1.8 times higher than that of the incident ultrasound. Prediction of further reflection is difficult, but a standing wave ratio given by a ratio of pressure amplitudes at antinode and nodes appearing on the coverslip surface might not be so high because reflection at a round-shape air-droplet boundary and non-uniform propagation path cause ultrasound interference in random phase. Z_m, Z_a, and Z_g are characteristic acoustic impedances of the medium, air, and glass, respectively. $R_{m/g}$ and $R_{m/a}$ are pressure reflection coefficients at medium-glass and medium-air boundaries.

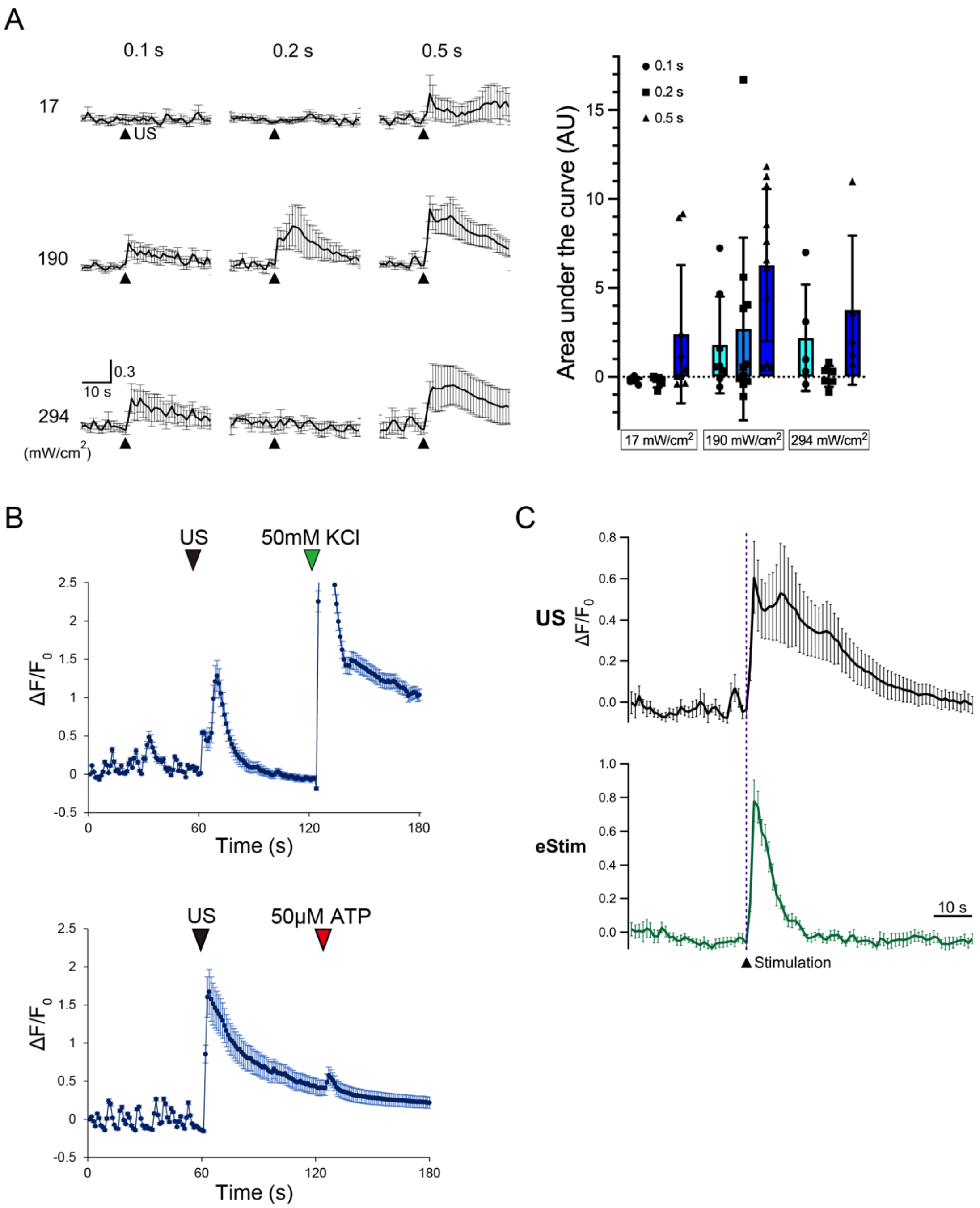

Fig. S2. Basic properties of ultrasound-induced Ca^{2+} transients of cultured mouse neurons

A) Ultrasound-induced neuronal responses and quantification of the responses as function of ultrasound intensity and stimulus duration. Stimulus intensities of 17, 190 and 294 mW/cm^2 were used, and they were stimulated for 0.1 (n = 6, 8, 5), 0.2 (n = 5, 11, 6), and 0.5 (n = 9, 11, 5) seconds, respectively. Arrowheads indicate the time point of ultrasound stimulation. The amplitude of Ca^{2+} transients in response to ultrasound stimulations is quantified as area under

the curve (AU). Traces and bar graph values show mean ± SEM. B) Neuronal viability and its healthy state after sonication were verified by application with 50 mM KCl (upper) and 50 μM ATP (lower). Representative averaged traces of neuronal Ca^{2+} transients against ultrasound irradiation in each condition. Data from three independent experiments are plotted as mean ± SEM, respectively. The arrowhead indicates the time of ultrasound stimulation (black), KCl (green), and ATP (red). C) Comparison of ultrasound-induced and electrical stimulation-induced neuronal calcium responses. Neuronal calcium responses against ultrasound irradiation (US) or electrical stimulation (eStim) of cultured neurons. Eleven and ten independent experiments for US and eStim, respectively, were performed. Trace values show mean ± SEM.

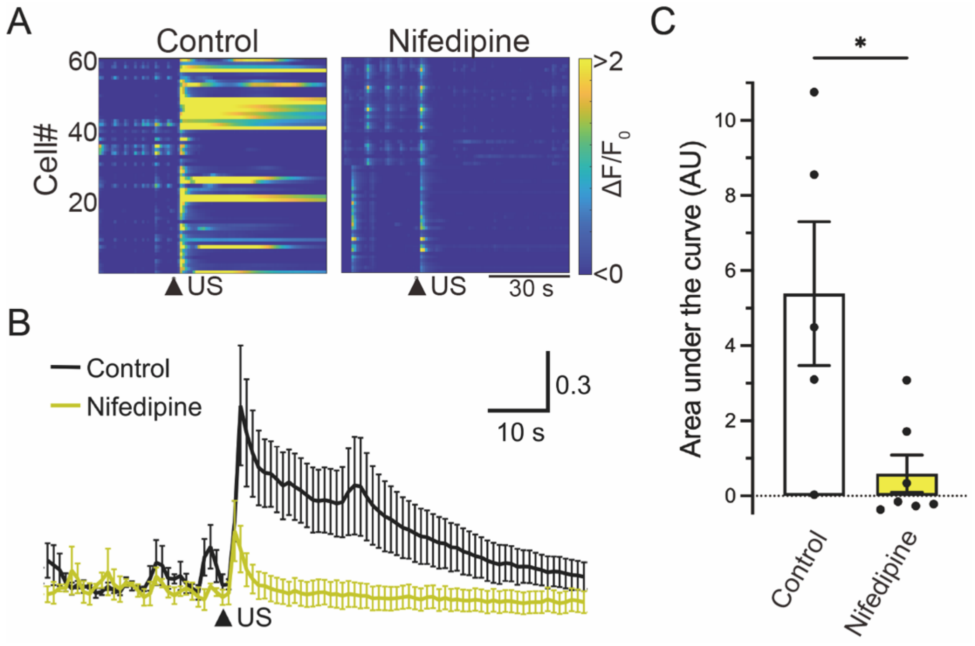

Fig. S3. Neuronal calcium responses induced by ultrasound irradiation in the presence of nifedipine

A) Heatmap demonstration of normalized fluorescence intensity ($\Delta F/F_0$) of GCaMP6s in neurons under the condition of control or the presence of nifedipine (100 μM). Nifedipine is used to block L-type voltage-gated calcium channels. Arrowheads indicate the time of ultrasound stimulation. B) Averaged traces of neuronal Ca^{2+} transients against ultrasound irradiation under the condition of control (black), or the presence of nifedipine (yellow). Data from five and seven independent experiments for control and nifedipine, respectively, are described by mean ± SEM. C) Bar graph values of area under the curve in each condition show mean ± SEM. *$P < 0.05$ (Unpaired *t* test).

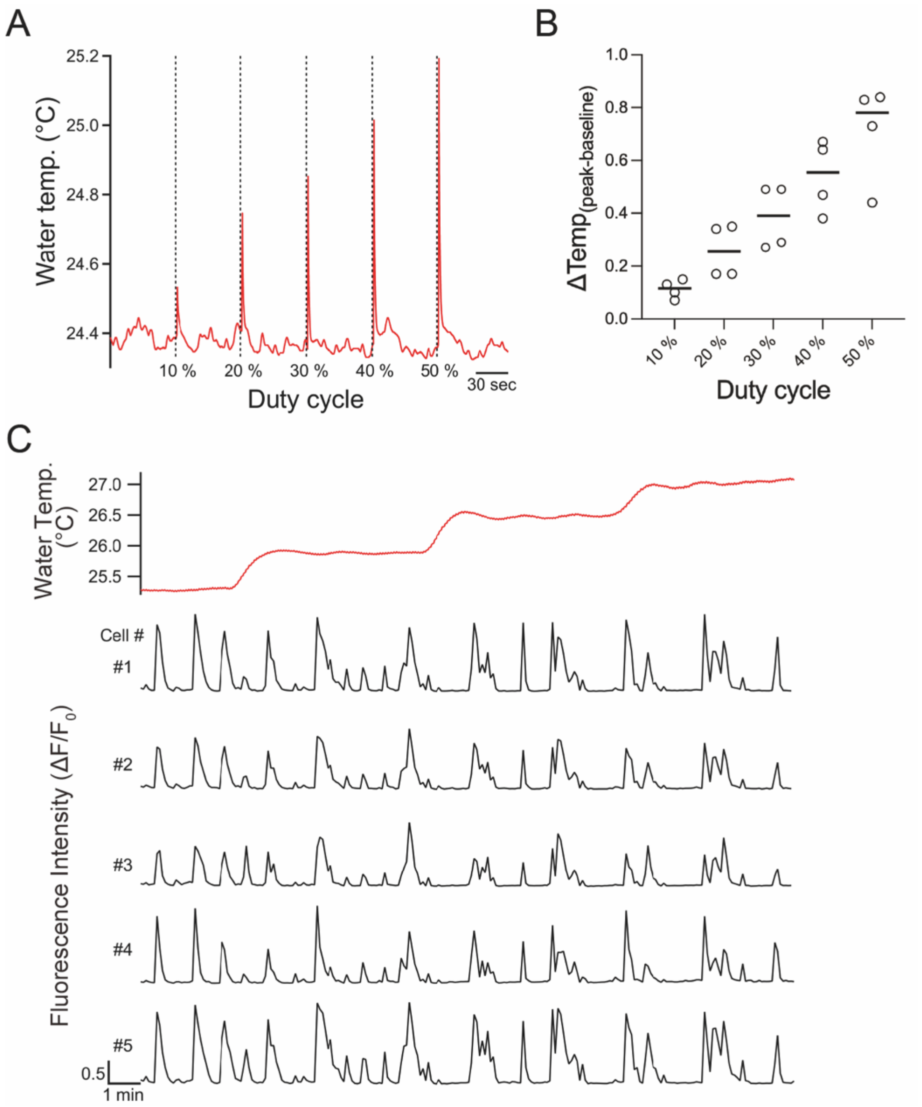

Fig. S4. Assessment of thermal effect

A) Ultrasound-induced temperature rise in bath solution was measured by a thermocouple probe. Bath temperatures were monitored in real-time during the sonication with the 10%, 20%, 30%, 40%, and 50% duty cycles, respectively. The dotted lines indicate the time point of ultrasound irradiation. B) $\Delta Temp = Temp_{(peak)} - Temp_{(baseline)}$ in the responses to sonication with various duty cycles are plotted. Baseline temperature $Temp_{(baseline)}$ was calculated as the average bath temperature for 10 sec during the pre-stimulation period. Data from four independent experiments are shown as mean ± SEM, respectively. C) Representative traces of spontaneous neuronal Ca^{2+} oscillation during the stepwise increase of bath temperature in individual neurons. The temperature of the bath solution was measured by thermocouple probe and plotted simultaneously.

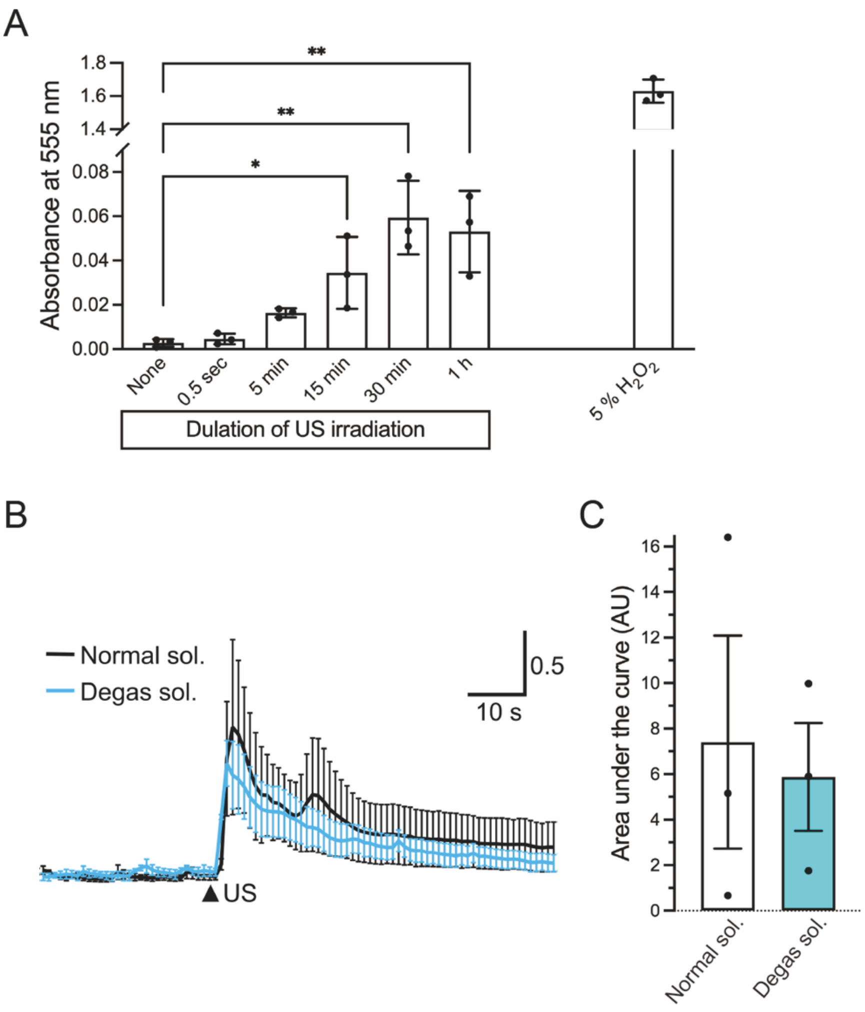

Fig. S5. Assessment of ultrasound-included cavitation activity

A) Measurement of cavitation-mediated free radical generation using a starch-iodine method. Data from three independent experiments for non-treated (None) and ultrasound-exposed samples. A sample treated with 5% H_2O_2 is demonstrated as a positive control of the assay reaction. Bar graph values in each condition show mean ± SEM. *$P < 0.05$, **$P < 0.01$ (one-way ANOVA followed by Dunnett *post-hoc* test). B) Calcium imaging of cultured neurons in both standard and degassed bath solutions. Note that ultrasound stimuli consistently induced similar levels of neuronal Ca^{2+} transient in both conditions. C) Bar graph values of area under the curve in each condition show mean ± SEM (Unpaired *t*-test).

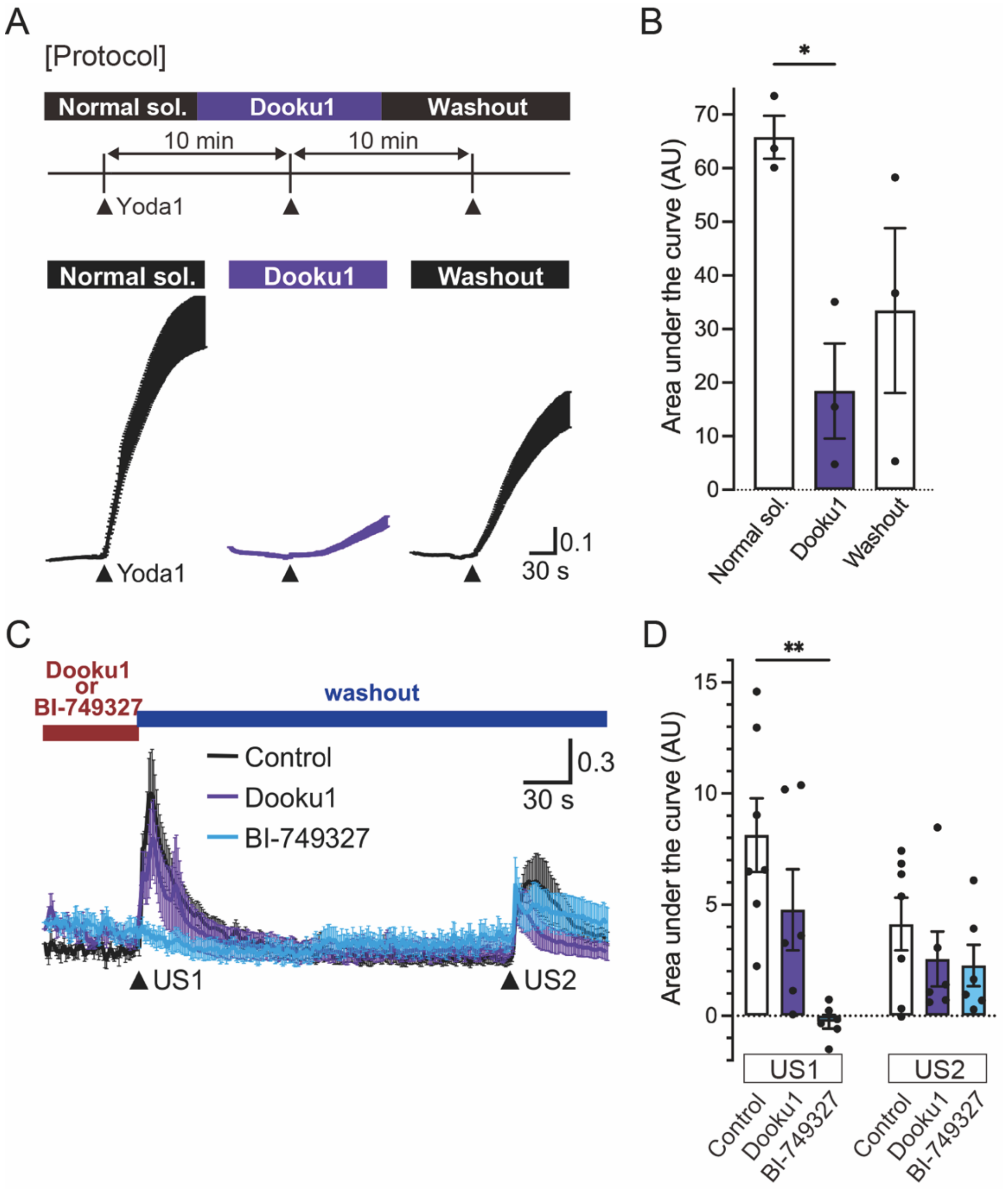

Fig. S6. Washout efficacy of Dooku1 and BI-749327

A, B) Yoda1-induced elevation of intracellular calcium concentration in N2a cells via activation of endogenous Piezo1. A) N2a cells expressing GCaMP6s were sequentially treated with 5 mM Yoda1 in the absence and presence of Dooku1 every 10 minutes, respectively. Note that N2a cells recovered the ability to respond against Yoda1 administration after the washout of Dooku1. Representative averaged traces of Ca^{2+} transients in response to Yoda1 treatment are shown. Data from 56−83 cells in each experiment were plotted as mean ± SEM, respectively. B) Bar graph values of area under the curve from three independent experiments show mean ± SEM. *$P < 0.05$ (one-way ANOVA followed by Dunnett *post-hoc* test). C) Averaged traces of neuronal Ca^{2+} transients against ultrasound stimulation under the condition of control (black, N=7), or the presence of Dooku1 (purple, N=6) or BI-749327 (light blue, N=6). 2nd ultrasound stimuli were

applied to induce neuronal responses after extensive washout of the compounds. Note that the washout of BI-749327 significantly restores the neuronal ability to respond against ultrasound. By contrast, neurons treated with Dooku1 show similar response to ultrasound before and after the washout, as do neurons in the control condition. Averaged traces show mean ± SEM; arrowhead indicates the time of ultrasound stimulation. D) Bar graph values of area under the curve in each condition show mean ± SEM. **$P < 0.01$ (one-way ANOVA followed by Dunnett *post-hoc* test).

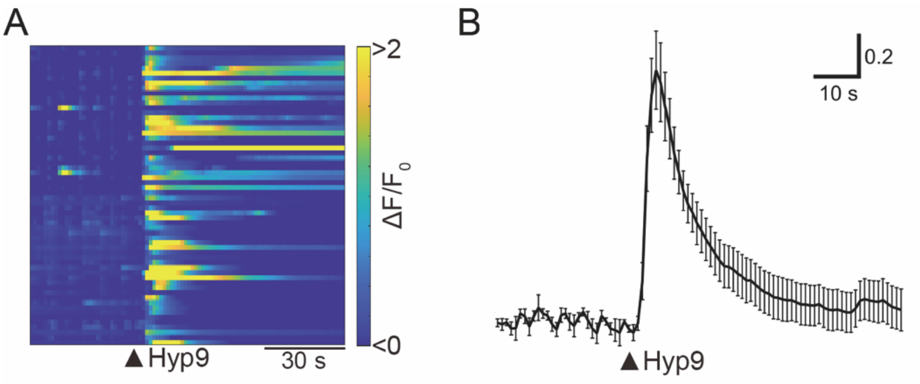

Fig.S7. Neuronal responses to a selective TRPC6 agonist, Hyp9

A) Heatmap demonstration of normalized fluorescence intensity ($\Delta F/F_0$) of neuronal responses when Hyp9 (10 μM) is applied. Arrowheads indicate the time of Hyp9 application. Data from 60 cells in two experiments are plotted. B) Averaged neuronal response to Hyp9 (n = 3).

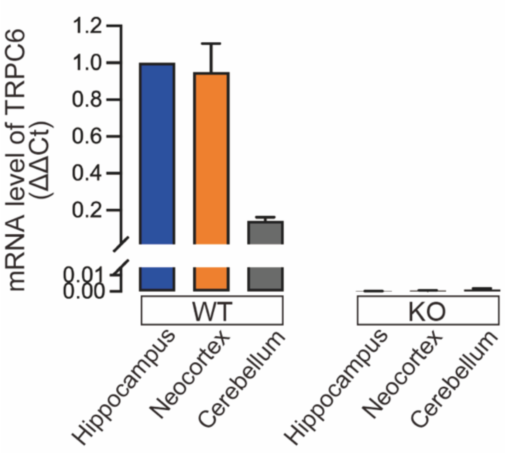

Fig. S8. qPCR validation of TRPC6 expression profile in WT or TRPC6-KO mouse brain

Normalized mRNA levels of TRPC6 against beta-actin (ΔΔCt) in hippocampus (blue), neocortex (orange), and cerebellum (gray) of WT or TRPC6-KO mice are shown. Bars show mean ± SD.

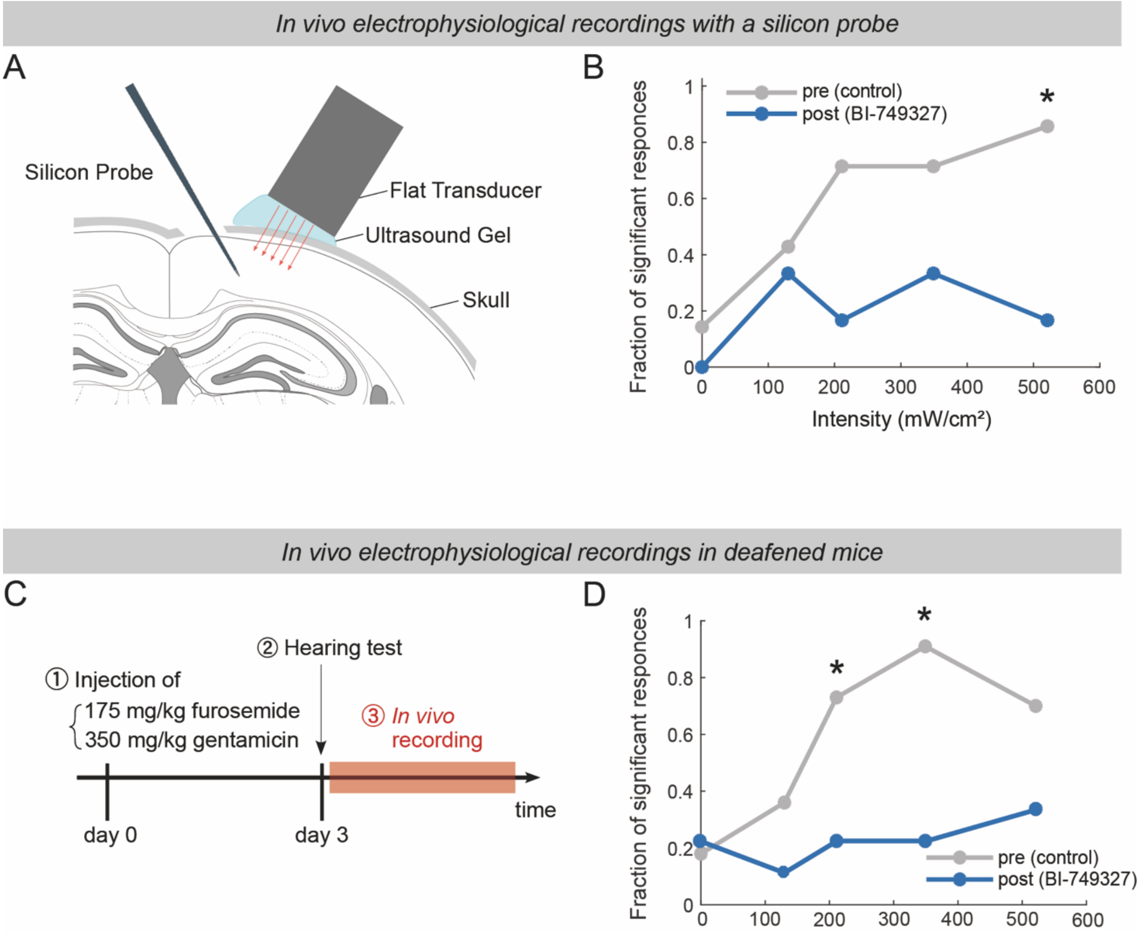

Fig. S9. Validation of TRP6C-mediated ultrasound neuromodulation *in vivo*

A, B) US intensity-dependent neuromodulation of cortical neurons *in vivo* and reduction of the modulation with a TRPC6 blocker, BI-749327. Note that the neural responses were recorded with 15 micron-thick silicon probes, which have been shown to be free of electrode-vibration-mediated artifacts. n = 35, 30 sessions from three mice for pre and post recordings, respectively. C, D) US intensity-dependent neuromodulation of cortical neurons *in vivo* and the reduction of the modulation with a TRPC6 blocker, BI-749327, in chemically deafened mice, where auditory confounding does not exist. n = 52, 42 sessions from three mice for pre and post recordings, respectively. Significance for each stimulus intensity in B and D was determined by X^2 test on a two-by-two (significance x control or BI-749327) frequency table. $^*P < 0.05$.

References

1. A. R. Nickolls *et al.*, Reevaluation of Piezo1 as a gut RNA sensor. *Elife* **11**, e83346 (2022).

2. K. Okada, N. Kudo, M. A. Hassan, T. Kondo, K. Yamamoto, Threshold curves obtained under various gaseous conditions for free radical generation by burst ultrasound - Effects of dissolved gas, microbubbles and gas transport from the air. *Ultrason Sonochem* **16**, 512-518 (2009).

3. Y. Yuan, A. Long, Y. Wu, X. Li, Closed-loop transcranial ultrasound stimulation with a fuzzy controller for modulation of motor response and neural activity of mice. *J Neural Eng* **19,** 036046 (2022).

4. S. L. McGuinness, R. K. Shepherd, Exogenous BDNF rescues rat spiral ganglion neurons in vivo. *Otol Neurotol* **26**, 1064-1072 (2005).

5. S. Imamura, J. C. Adams, Changes in cytochemistry of sensory and nonsensory cells in gentamicin-treated cochleas. *J Assoc Res Otolaryngol* **4**, 196-218 (2003).
